# Eating disorder outcomes: findings from a rapid review of over a decade of research

**DOI:** 10.1186/s40337-023-00801-3

**Published:** 2023-05-30

**Authors:** Jane Miskovic-Wheatley, Emma Bryant, Shu Hwa Ong, Sabina Vatter, Anvi Le, National Eating Disorder Research Consortium, Phillip Aouad, Sarah Barakat, Robert Boakes, Leah Brennan, Emma Bryant, Susan Byrne, Belinda Caldwell, Shannon Calvert, Bronny Carroll, David Castle, Ian Caterson, Belinda Chelius, Lyn Chiem, Simon Clarke, Janet Conti, Lexi Crouch, Genevieve Dammery, Natasha Dzajkovski, Jasmine Fardouly, Carmen Felicia, John Feneley, Amber-Marie Firriolo, Nasim Foroughi, Mathew Fuller-Tyszkiewicz, Anthea Fursland, Veronica Gonzalez-Arce, Bethanie Gouldthorp, Kelly Griffin, Scott Griffiths, Ashlea Hambleton, Amy Hannigan, Mel Hart, Susan Hart, Phillipa Hay, Ian Hickie, Francis Kay-Lambkin, Ross King, Michael Kohn, Eyza Koreshe, Isabel Krug, Jake Linardon, Randall Long, Amanda Long, Sloane Madden, Sarah Maguire, Danielle Maloney, Peta Marks, Sian McLean, Thy Meddick, Jane Miskovic-Wheatley, Deborah Mitchison, Richard O’Kearney, Shu Hwa Ong, Roger Paterson, Susan Paxton, Melissa Pehlivan, Genevieve Pepin, Andrea Phillipou, Judith Piccone, Rebecca Pinkus, Bronwyn Raykos, Paul Rhodes, Elizabeth Rieger, Sarah-Catherine Rodan, Karen Rockett, Janice Russell, Haley Russell, Fiona Salter, Susan Sawyer, Beth Shelton, Urvashnee Singh, Sophie Smith, Evelyn Smith, Karen Spielman, Sarah Squire, Juliette Thomson, Stephen Touyz, Ranjani Utpala, Lenny Vartanian, Sabina Vatter, Andrew Wallis, Warren Ward, Sarah Wells, Eleanor Wertheim, Simon Wilksch, Michelle Williams, Stephen Touyz, Sarah Maguire

**Affiliations:** 1 Faculty of Medicine and Health, InsideOut Institute for Eating Disorders, University of Sydney, Level 2, Charles Perkins Centre (D17), Sydney, NSW 2006 Australia; 2 Sydney Local Health District, Sydney, Australia; 3 Healthcare Management Advisors, Melbourne, Australia; 4 School of Psychology, Faculty of Science, University of Sydney, Sydney, NSW Australia; 5 School of Psychology and Public Health, La Trobe University, Victoria, Australia; 6 School of Psychology, Perth, Western Australia Australia; 7 Eating Disorders Victoria, Victoria, Australia; 8 Perth, Australia; 9 Medicine, Dentistry and Health Sciences, University of Melbourne, Victoria, Australia; 10 School of Life and Environmental Sciences, University of Sydney, Sydney, NSW Australia; 11 Eating Disorders Queensland, Brisbane, QLD Australia; 12 Sydney Local Health District, New South Wales Health, Sydney, Australia; 13 Westmead Hospital, Sydney, NSW Australia; 14 Translational Health Research Institute, Western Sydney University, Sydney, NSW Australia; 15 Brisbane, Australia; 16 School of Psychology, University of New South Wales, Sydney, NSW Australia; 17 University of Sydney, Sydney, NSW Australia; 18 New South Wales Health, Sydney, NSW Australia; 19 School of Psychology, Faculty of Health, Deakin University, Victoria, Australia; 20 School of Population Health, Faculty of Health Sciences, Curtain University, Perth, Australia; 21 Hollywood Clinic, Ramsay Health Care, Perth, Australia; 22 Melbourne School of Psychological Sciences, University of Melbourne, Victoria, Australia; 23 Queensland Eating Disorder Service, Brisbane, QLD Australia; 24 Hunter New England Local Health District, New Lambton, NSW Australia; 25 St Vincent’s Hospital Network Local Health District, Sydney, NSW Australia; 26 Brain and Mind Centre, University of Sydney, Sydney, Australia; 27 School of Medicine and Public Health, University of Newcastle, Newcastle, NSW Australia; 28 Westmead Hospital, Sydney, Australia; 29 College of Medicine and Public Health, Flinders University, Adelaide, SA Australia; 30 Exchange Consultancy, Redlynch, NSW Australia; 31 Eating Disorders Service, Children’s Hospital at Westmead, Sydney, NSW Australia; 32 The Bouverie Centre, School of Psychology and Public Health, La Trobe University, Victoria, Australia; 33 Clinical Excellence Queensland, Mental Health Alcohol and Other Drugs Branch, Brisbane, QLD Australia; 34 College of Health and Medicine, Australian National University, Canberra, ACT Australia; 35 ADHD and BED Integrated Clinic, Melbourne, VIC Australia; 36 Department of Psychology and Counselling, La Trobe University, Victoria, Australia; 37 School of Health and Social Development, Faculty of Health, Deakin University, Geelong, VIC Australia; 38 Swinburne Anorexia Nervosa (SWAN) Research Group, Centre for Mental Health, School of Health Sciences, Swinburne University, Victoria, Australia; 39 Children’s Health Queensland Hospital and Health Service, Brisbane, QLD Australia; 40 Centre for Clinical Interventions, Western Australia Health, Perth, WA Australia; 41 Central Clinical School Brain & Mind Research Institute, University of Sydney, Sydney, NSW Australia; 42 Ramsay Health Care, Perth, Australia; 43 Department of Paediatrics, The University of Melbourne, Parkville, Australia; 44 National Eating Disorders Collaboration, Victoria, Australia; 45 The Hollywood Clinic Hollywood Private Hospital, Ramsey Health, Perth, Australia; 46 Sydney, Australia; 47 The Butterfly Foundation, Sydney, Australia; 48 Eating Disorder Service, The Sydney Children’s Hospital Network, Westmead Campus, Sydney, Australia; 49 Department of Psychiatry, University of Queensland, Brisbane, Australia; 50 University of Tasmania, Hobart, TAS Australia; 51 College of Education, Psychology and Social Work, Flinders University, Adelaide, SA Australia; 52 Royal Hobart, Tasmanian Health Service, Hobart, TAS Australia

**Keywords:** Eating disorders, Anorexia nervosa, Bulimia nervosa, Binge eating disorder, Outcomes, Transdiagnostic, Remission, Recovery, Relapse, Mortality

## Abstract

**Background:**

Eating disorders (ED), especially Anorexia Nervosa (AN), are internationally reported to have amongst the highest mortality and suicide rates in mental health. With limited evidence for current pharmacological and/or psychological treatments, there is a grave responsibility within health research to better understand outcomes for people with a lived experience of ED, factors and interventions that may reduce the detrimental impact of illness and to optimise recovery. This paper aims to synthesise the literature on outcomes for people with ED, including rates of remission, recovery and relapse, diagnostic crossover, and mortality.

**Methods:**

This paper forms part of a Rapid Review series scoping the evidence for the field of ED, conducted to inform the Australian National Eating Disorders Research and Translation Strategy 2021–2031, funded and released by the Australian Government. ScienceDirect, PubMed and Ovid/MEDLINE were searched for studies published between 2009 and 2022 in English. High-level evidence such as meta-analyses, large population studies and Randomised Controlled Trials were prioritised through purposive sampling. Data from selected studies relating to outcomes for people with ED were synthesised and are disseminated in the current review.

**Results:**

Of the over 1320 studies included in the Rapid Review, the proportion of articles focused on outcomes in ED was relatively small, under 9%. Most evidence was focused on the diagnostic categories of AN, Bulimia Nervosa and Binge Eating Disorder, with limited outcome studies in other ED diagnostic groups. Factors such as age at presentation, gender, quality of life, the presence of co-occurring psychiatric and/or medical conditions, engagement in treatment and access to relapse prevention programs were associated with outcomes across diagnoses, including mortality rates.

**Conclusion:**

Results are difficult to interpret due to inconsistent study definitions of remission, recovery and relapse, lack of longer-term follow-up and the potential for diagnostic crossover. Overall, there is evidence of low rates of remission and high risk of mortality, despite evidence-based treatments, especially for AN. It is strongly recommended that research in long-term outcomes, and the factors that influence better outcomes, using more consistent variables and methodologies, is prioritised for people with ED.

**Supplementary Information:**

The online version contains supplementary material available at 10.1186/s40337-023-00801-3.

## Abstract (plain language summary)

Eating disorders are complex psychiatric conditions that can seriously impact a person’s physical health. Whilst they are consistently associated with high mortality rates and significant psychosocial difficulties, lack of agreement on definitions of recovery, remission and relapse, as well as variations in methodology used to assess for standardised mortality and disability burden, means clear outcomes can be difficult to report. The current review is part of a larger Rapid Review series conducted to inform the development of Australia’s National Eating Disorders Research and Translation Strategy 2021–2031. A Rapid Review is designed to comprehensively summarise a body of literature in a short timeframe to guide policymaking and address urgent health concerns. This Rapid Review synthesises the current evidence-base for outcomes for people with eating disorders and identifies gaps in research and treatment to guide decision making and future clinical research. A critical overview of the scientific literature relating to outcomes in Western healthcare systems that may inform health policy and research in an Australian context is provided in this paper. This includes remission, recovery and relapse rates, diagnostic cross-over, the impact of relapse prevention programs, factors associated with outcomes, and findings related to mortality.

**Supplementary Information:**

The online version contains supplementary material available at 10.1186/s40337-023-00801-3.

## Introduction

Eating disorders (ED), especially Anorexia Nervosa (AN), have amongst the highest mortality and suicide rates in mental health. While there has been significant research into causal and maintaining factors, early identification efforts and evidence-based treatment approaches, global incidence rates have increased from 3.4% calculated between 2000 and 2006 to 7.8% between 2013 and 2018 [1]. While historically seen as a female illness, poorer outcomes are increasingly seen in other genders, including males [2].

Over 3.3 million healthy life years are lost worldwide due to ED each year, and many more lost to disability due to medical and psychiatric complications [3]. Suicide accounts for approximately 20% of non-natural deaths among people with ED [4]. As this loss of healthy life is preventable, there is a grave responsibility to better understand outcomes for people with ED, including factors which may minimise the detrimental impact they have on individuals, carers, and communities, as well as to optimise recovery.

There has been considerable debate within the clinical, scientific and lived experience (i.e., patient, consumer, carer) communities about the definition and measurement of key outcomes in ED, including ‘remission’ from illness (a period of relief from symptoms), ‘relapse’ (a resumption of symptoms) and ‘recovery’ (cessation of illness) [5, 6], which can compromise outcome comparisons. Disparities include outcome variables relating to eating behaviours as well as medical, psychological, social and quality of life factors. There is increasing awareness in the literature of the elevated likelihood of diagnostic crossover [7]; research examining specific diagnostic profiles potentially misses outcomes where symptom experience transforms rather than alleviates. Methodological approaches in outcomes research are varied, the most significant being length of time to follow up, compromising direct study comparisons.

The aim of this Rapid Review (RR) is to synthesise the literature on outcomes for people with ED, including rates of remission, recovery and relapse, diagnostic crossover, and mortality. Factors influencing outcomes were summarised including demographic, illness, treatment, co-morbidities, co-occurring health conditions, societal factors, and impact of relapse prevention programs. This RR forms one of a series of reviews scoping the field of ED commissioned to inform the Australian National Eating Disorders Research and Translation Strategy 2021–2031 [8]. The objective is to evaluate the current literature in ED outcomes to identify areas of consensus, knowledge gaps and suggestions for future research.

## Methods

The Australian Government Commonwealth Department of Health funded the InsideOut Institute for Eating Disorders (IOI) to develop the Australian Eating Disorders Research and Translation Strategy 2021–2031 [8] under the Psych Services for Hard to Reach Groups initiative (ID 4-8MSSLE). The strategy was developed in partnership with state and national stakeholders including clinicians, service providers, researchers, and experts by lived experience (including consumers and families/carers). Developed through a 2 year national consultation and collaboration process, the strategy provides the roadmap to establishing ED as a national research priority and is the first disorder-specific strategy to be developed in consultation with the National Mental Health Commission. To inform the strategy, IOI commissioned Healthcare Management Advisors (HMA) to conduct a series of RRs to broadly assess all available peer-reviewed literature on the six DSM-V [9] listed ED. RR’s were conducted in the following domains: (1) population, prevalence, disease burden, Quality of Life in Western developed countries; (2) risk factors; (3) co-occurring conditions and medical complications; (4) screening and diagnosis; (5) prevention and early intervention; (6) psychotherapies and relapse prevention; (7) models of care; (8) pharmacotherapies, alternative and adjunctive therapies; and (9) outcomes (including mortality) (current RR), with every identified paper allocated to only one of the above domains from abstract analysis by two investigators. Each RR was submitted for independent peer review to the *Journal of Eating Disorders* special edition, “Improving the future by understanding the present: evidence reviews for the field of eating disorders”.

A RR Protocol [10] was utilised to swiftly synthesise evidence to guide public policy and decision-making [11]. This approach has been adopted by several leading health organisations, including the World Health Organization [12] and the Canadian Agency for Drugs and Technologies in Health Rapid Response Service [13], to build a strong evidence base in a timely and accelerated manner, without compromising quality. RR was chosen as the most suitable design as it is conducted with broader search terms and inclusion criteria allowing to gain a better understanding of a specific field, returning a larger number of search results and providing a snapshot of key findings detailing the current state of a field at study [10]. A RR is not designed to be as comprehensive as a systematic review—it is purposive rather than exhaustive and provides actionable evidence to guide health policy [14].

The RR is a narrative synthesis adhering to the PRISMA guidelines [15]. It is divided by topic area and presented as a series of papers. Three research databases were searched: ScienceDirect, PubMed and Ovid/MEDLINE. To establish a broad understanding of the progress made in the field of eating disorders, and to capture the largest evidence base on the past 13 years (originally 2009–2019, but expanded to include the preceding two years), the eligibility criteria for included studies into the RR were kept broad. Therefore, included studies were published between 2009 and 2022, in English, and conducted within Western healthcare systems or health systems comparable to Australia in terms of structure and resourcing. The initial search and review process was conducted by three reviewers between 5 December 2019 and 16 January 2020. The re-run for the years 2020–2021 was conducted by two reviewers at the end of May 2021 and a final run for 2022 conducted in January 2023 to ensure the most up to date publications were included prior to publication.

The RR had a translational research focus with the objective of identifying evidence relevant to developing optimal care pathways. Searches, therefore, used a Population, Intervention, Comparison, Outcome (PICO) approach to identify literature relating to population impact, prevention and early intervention, treatment, and long-term outcomes. Purposive sampling focused on high-level evidence studies such as: meta-analyses; systematic reviews; moderately sized randomised controlled trials (RCTs) (*n* > 50); moderately sized controlled-cohort studies (*n* > 50), or population studies (*n* > 500). However, the diagnoses Avoidant Restrictive Food Intake Disorder (ARFID), Eating Disorder Not Otherwise Specified (EDNOS), Other Specified Feeding or Eating Disorder (OSFED) and Unspecified Feeding or Eating Disorder (UFED) necessitated a less stringent eligibility criterion due to a paucity of published articles. As these diagnoses are newly captured in the DSM-V [9] (released in 2013, within the allocated search timeframe), the evidence base is emerging, and fewer studies have been conducted. Thus, smaller studies (*n* ≤ 20) and narrative reviews were also considered and included. Grey literature, such as clinical or practice guidelines, protocol papers (without results) and Masters’ theses or dissertations, was excluded.

Full methodological details including eligibility criteria, search strategy and terms and data analysis are published in a separate protocol paper [10]. The full RR included a total of over 1320 studies (see Additional file 1: Fig. S1). Data from included studies relating to outcomes for eating disorders were synthesised and are presented in the current review.

## Results

Of the 1320 articles included in the RR, the proportion of articles focused on outcomes in ED was relatively small, just less than 9% (*n* = 116) (see Additional file 2: Table S1). Studies typically examined outcomes in AN, Bulimia Nervosa (BN) and Binge Eating Disorder (BED), with limited research in other diagnostic groups. Whereas most outcome studies reported recovery, remission and relapse rates, others explored factors impacting outcomes, such as quality of life, co-occurring conditions, and outcomes from relapse prevention programs.

ED, particularly AN, have long been associated with an increased risk of mortality. The current review summarises best available evidence exploring this association. Several factors complicate these findings including a lack of consensus on definitions of remission, recovery and relapse, widely varying treatment protocols and research methodologies, and limited transdiagnostic outcome studies or syntheses such as meta-analyses. Table 1 provides a summary of outcomes reported by studies identified in this review. There is considerable heterogeneity in the reported measures.

**Table 1:** Summary of patient outcomes including predictors and mediators by eating disorder diagnosis

ED	Outcome—recovery/remission	Predictors/mediators
AN (full recovery, weight restored)	Overall 21.8% [16]–70.1% [17] Follow-up 17.8% [18]–62.8% [19] 60.3% [20] Transdiagnostic From AN to EDNOS: 33.0% [21] From AN to BN: 23.4% [7] From BN to AN: 8.4% [7]	**Predicted good outcomes:** AN-R over AN-BP Younger age at treatment No pharmacotherapy Shorter duration of illness Higher baseline BMI
BN (binge/purge abstinence)	Post-treatment [22] 35.4% (completers) 29.9% (intent to treat) Follow-up [22] 34.6% (completers) 28.6% (intent to treat)	**Predicted good treatment response:** Low levels of shape/weight concern Less severe depressive symptoms
BED (binge abstinence)	Post-treatment [22] 50.9% to (completers) 50.3% (intent to treat) Follow-up [22] 45.1% (completers) 42.3% (intent to treat)	**Predicted good outcomes:** Rapid behavioural and cognitive change in response to treatment Predicted poor outcomes Severity of obesity
ARFID (mean %MBMI weight restoration, no DSM diagnosis)	Post-treatment 94% (within 95% median BMI) [23] Follow-up 95% (within 95% median BMI) [23] 47.4% [24]–62% [25] no psychiatric diagnosis	**None identified**
Any ED* (no longer meeting criteria, levels of psychosocial function)	Child and adolescent: 23.0% [26] Young adult: 89.0% [27] Mid-aged: 80.0% [21]	**Predicted good outcomes:** Rapid behavioural and cognitive change in response to treatment

NB—Figures reported in the table for BN and BED are results from systematic review, which differentiated between treatment completer and intent to treat analysis [22]

*Any ED covers Feeding and/or Eating Disorders Not Otherwise Specified, OSFED

### Overall outcomes

A *good* outcome for a person experiencing ED symptomatology is commonly defined as either remission or no longer meeting diagnostic criteria, as well as improved levels of psychosocial functioning and quality of life [28, 29]. However, such a comprehensive approach is rarely considered, and there is no consensus on a definition for recovery, remission, or relapse for any of the ED diagnoses [30, 31]. To contextualise this variation, definitions and determinants for these terms are presented in Table 2.

**Table 2:** Sample of definitions of relapse, remission, and recovery in eating disorder research

References	Diagnostic group/s	Assessment period	Determinant/s	Definitions
*Relapse*				
Agras et al. [21]	ED	Every 3 months to 4 years	EDE^a^	Symptoms for any ED diagnosis determined by EDE^a^
Castellini et al. [7]	ED	Every 3 years to 6 years	Diagnosis	Return to a full syndromal or EDNOS criteria after a period of remission [32]
Stice et al. [33]	ED	Every year to 8 years	Diagnosis	Meeting criteria after ≥ 1 month recovery [34]
Carter et al. [35]	AN	1 year	BMI or behaviours	BMI ≤ 17.5 (3 months) or at least one episode of binge-purge behaviour per week (3 months)
Berends et al. [36]	AN	1.5 years	BMI & diagnosis	Full: BMI < 18.5 for adults and BMI < − 1 SD for adolescents and full recurrence of core diagnostic symptoms (DSM-IV) assessed by clinical team Partial: Re-occurrence of core diagnostic symptoms (DSM-IV) assessed by clinical team
Le Grange et al. [30]	AN	Every year to 4 years	%EBW^b^	< 87% EBW^b^
*Remission*				
Walker et al. [37]	ED	1 week	EAT^c^	Within 1 SD of community norms on the EAT^c^ [38]
Custal et al. [39]	ED	4 weeks	Behaviours & psychology	Full: Absence of binging and purging (laxatives and/or vomiting) behaviours (1 month) and psychological improvement measured by clinical questionnaire Partial: Substantial symptomatic improvement but presence of residual symptoms (reduction of at least 50% of bulimic symptoms) [40]
Fernández-Aranda et al. [41]	ED	16 weeks	Diagnosis	Full: Absence of diagnostic symptoms (DSM-V) (1 month) assessed by clinical team Partial: Substantial symptomatic improvement but with residual symptoms assessed by clinical team
Colton et al. [42]	ED	1 year	Diagnosis or behaviours	No reported disturbed eating behaviour or absence of diagnostic symptoms
Tomba et al. [43]	ED	1 year	Diagnosis, BMI, behaviours, EAT^c^	Absence of diagnosis (DSM-IV-TR), BMI, absence of binge-eating, purging, or fasting (3 month), EAT^c^ < 30 [28]
Johnston et al. [26]	ED	Every 6 months to 1 year	EDE^a^ & diagnosis	Absence of diagnostic criteria as determined by the EDE^a^ and clinical team at 12 month review
Helverskov et al. [44]	ED	2.5 years	Symptoms	Full: Absence of symptoms or the presence of only residual symptoms (3 months) Partial: Reduction of symptoms to a sub-diagnostic level (3 months)
Agras et al. [21]	ED	Every 3 months to 4 years	EDE^a^	Absence of ED diagnosis (6 months) determined by EDE^a^
Stice et al. [27]	ED	Every year for 8 years	Diagnosis	Absence of diagnosis (1 month) [34]
Quadflieg et al. [45]	ED	22 years (average 11 years)	Diagnosis	Absence of diagnosis (DSM IV) (3 months)
Brown et al. [18]	AN	0.5 years	%EBW^b^ & EDE & behaviours	> 95% EBW^b,^ no fasting or binge eating/purging within the past month, and EDE-Q global score within 1 SD of adolescent norms [46]
Wade et al. [47]	AN	1 year	Behaviours & EDE-Q^d^	Absence of eating disorder behaviours (binge eating, purging, driven exercise, and fasting) (3 months) and normative levels of eating disorder psychopathology (1 month) determined by EDE-Q^d^ within 1 SD of community norms [48]
Strandjord et al. [25]	ARFID & AN	1 year	Diagnosis & %EBW^b^	> 90% EBW and absence of diagnosis (3 months)
Le Grange et al. [30]	AN	Every year to 4 years	%EBW^b^ & EDE^a^	> 95% EBW and EDE^a^ global score within 1 SD of community norms
Franko et al. [49]	AN, BN	Every 6 months to 25 years	Diagnosis	Absence of diagnostic criteria (DSM-5) for a sustained period of time [50]
Gorrell et al. [31]	BN	Every 6 months to 1 year	Diagnosis, behaviours & EDE^a^	Model A: Absence from eight compensatory weight control behaviours (DSM-5) that contribute to BN Model B: Model A and EDE^a^ global scores within 1SD of community norms (i.e., < 3.05; Alison, 1996) Model C: Absence from objective and subjective binge eating and self-induced vomiting plus EDE^a^ global scores within 1SD of community norms
Lydecker et al. [51]	BED	0.5 years	Behaviours	Absence of binge-eating episodes for 4 consecutive weeks
*Recovery*				
Bardone-Cone et al. [29]	ED	3 months	Diagnosis, BMI, behaviours & EDE-Q^d^	Full: Absence of diagnosis (3 months), absence of behaviours (bingeing, purging, or fasting) (3 months), BMI > 18.5, EDE-Q^d^ sub scale scores within 1SD of age-matched community norms Partial: Absence of physical and behavioural symptoms, but persistence of psychological symptoms
Castellini et al. [7]	ED	Every 3 years for 6 years	Diagnosis	Absence of diagnosis (DSM-IV or DSM-V) (3 years) [32]
Stice et al. [33]	ED	Every year for 8 years	Diagnosis	Absence of diagnosis (1 month) [34]
Eielsen et al. [52]	ED	17 years	EDE^a^	EDE^a^ global score [53, 54]
Wild et al. [55]	AN	1 year	Diagnosis	Absence of diagnosis (12 months)
Zerwas et al. [56]	AN	1 year	Diagnosis	Absence of diagnosis (12 months)
Ricca et al. [57]	AN, subthreshold-AN	3 years	Diagnosis	Absence of diagnosis (DSM-IV)
Winkler et al. [58]	AN	12 years	Diagnosis	Absence of diagnosis (12 months)
Rigaud et al. [20]	AN	Yearly for 13 years	BMI, behaviours, psychology	BMI ≥ 18.5, absence of excessive physical exercising, normalised eating, no reported obsessive body shape or weight concerns
Wentz et al. [59]	AN	18 years (at 2, 6, 8, 12, 16 years)	Diagnosis	Absence of diagnosis (6 months)
Castellini et al. [7]	AN, BN (Binge & Purging subtype)	6 years	Diagnosis	Absence of diagnosis (DSM-IV) (8 weeks)
Franko et al. [49]	AN, BN	Every 6 months for 25 years	PSR^e^	PSR^e^ ≤ 2 at end of 52 week data collection period [50]
Castellini et al. [60]	BED & BN	3 years	Diagnosis	Absence of diagnosis (DSM-IV)

NB—Ordered by diagnostic group then follow-up period. Where authors referred to definitions from a previous study, references included

^a^*EDE* Eating disorder examination

^b^*%EBW* Percent expected body weight for age, gender, and height

^c^*EAT* Eating Attitudes Test (EAT-26)

^d^*EDE-Q* Eating Disorder Examination—Questionnaire

^e^*PSR* Psychiatric Status Rating

The terms ‘remission’ and ‘recovery’ appear to be used interchangeably in the literature. Whilst ‘remission’ is usually defined by an absence of diagnostic symptomatology, and ‘recovery’ an improvement in overall functioning, the period in which an individual must be symptom-free to be considered ‘remitted’ or ‘recovered’ varies greatly between studies, follow-up (FU) time periods are inconsistent, and very few studies examine return to psychosocial function and quality of life (QoL) after alleviation of symptoms. The current review uses the terms adopted by the original studies. ‘Relapse’ is typically defined by a return of symptoms after a period of symptom relief. The reviewed studies report a variety of symptom determinants including scores on standardised psychological and behavioural interviews or questionnaires, weight criteria [including Body Mass Index (BMI) or %Expected Body Weight (%EBW)], clinical assessment by a multidisciplinary team, self-reported ED behaviours, meeting diagnostic criteria, or a combination of the above.

#### Remission, recovery, and relapse

In a global overview of all studies reviewed, remission or recovery rates were reported for around half of the cohort, regardless of diagnostic group. For example, a 30 month FU study of a transdiagnostic cohort of patients found 42% obtained full and 72% partial remission, with no difference between diagnostic groups for younger people; however, bulimic symptoms emerged frequently during FU, regardless of initial diagnosis [44]. A 6 year study following the course of a large clinical sample (*n* = 793) reported overall recovery rates of 52% for AN, 50–52% for BN, 57% for EDNOS-Anorectic type (EDNOS-A), 60–64% for BED and 64–80% for EDNOS-Bulimic type (EDNOS-B) [7]. Of those who recorded full remission at end of treatment (EOT), relapse was highest for AN (26%), followed by BN (18%), and EDNOS-B (16%). Relapse was less common for individuals with BED (11–12%), and EDNOS-A (4%). Change in diagnosis (e.g., from AN to BN) was also seen within the relapse group [7].

Longer-term FU studies may more accurately reflect the high rates of relapse and diagnostic crossover associated with ED. A 17 year outcome study of ED in adult patients found only 29% remained fully recovered, with 21% partially recovered and half (50%) remaining ill [52], noting the protracted nature of illness for adults with longstanding ED. Relapse is observed at high rates (over 30%) among people with AN and BN at 22 year FU [61]. In a large clinical study using predictive statistical modelling, full remission was more likely for people with BED (47.4%) and AN (43.9%) compared to BN (25.2%) and OSFED (23.2%) [41]. This result is distinct from other studies citing AN to have the worst clinical outcomes within the diagnostic profiles [52]. The cut‐off points for the duration of illness associated with decreased likelihood of remission were 6–8 years for OSFED, 12–14 years for AN/BN and 20–21 years for BED [41]. As with recovery rates, reported rates of relapse are highly variable due to differing definitions and study methodologies used by researchers in FU studies [35, 61].

Evidence from a meta-analysis of 16 studies found four factor clusters that significantly contributed to relapse; however, also noted a substantial variability in procedures and measures compromising study comparison [62]. Factors contributing to heightened risk of relapse included severity of ED symptoms at pre- and post-treatment, presence and persistence of co-occurring conditions, higher age at onset and presentation to assessment, and longer duration of illness. Process treatment variables contributing to higher risk included longer duration of treatment, previous engagement in psychiatric and medical treatment (including specialist ED treatment) and having received inpatient treatment. These variables may indicate more significant illness factors necessitating a higher intensity of treatment.

Importantly, full recovery is possible, with research showing fully recovered people may be indistinguishable from healthy controls (HCs) on all physical, behavioural, and psychological domains (as evaluated by a battery of standardised assessment measures), except for anxiety (those who have fully recovered may have higher general anxiety levels than HCs) [29].

#### Diagnostic crossover

Most studies reported outcomes associated with specific ED diagnoses; however, given a significant proportion of individuals will move between ED diagnoses over time, it can be challenging to determine diagnosis-specific outcomes. Results from a 6 year FU study indicated that overall individuals with ED crossed over to other ED diagnoses during the FU observational period, most commonly AN to BN (23–27%), then BN to BED (8–11%), BN to AN (8–9%) and BED to BN (7–8%) [7]. Even higher crossover trends were observed in the subgroup reporting relapse during the FU period, with 61.5% of individuals originally diagnosed with AN developing BN, 27.2% and 18.1% of individuals originally diagnosed with BN developing AN and BED respectively, and 18.7% of people with a previous diagnosis of BED developing BN [7].

A review of 79 studies also showed a significant number of individuals with BN (22.5%) crossed over to other diagnostic groups (mostly OSFED) at FU [63]. A large prospective study of female adolescents and young adults in the United States (*n* = 9031) indicated that 12.9% of patients with BN later developed purging disorder and between 20 and 40% of individuals with subthreshold disorders progressed to full threshold disorders [64]. Progression from subthreshold to threshold eating disorders was higher for BN and BED (32% and 28%) than for AN (0%), with researchers suggesting higher risk for binge eating [66]. Progression from subthreshold to full threshold BN and BED was also common in adolescent females over the course of an 8 year observational study [33]. Some researchers contend that such diagnostic ‘instability’ demonstrates a need for ‘dimensional’ approaches to research and treatment which have greater focus on the severity rather than type of symptoms [7]. Diagnostic crossover is common and should be considered in the long-term management and monitoring of people with an ED.

#### Anorexia nervosa (AN)

People with restrictive-type ED have the poorest prognosis compared to the other diagnostic groups, particularly individuals displaying severe AN symptomatology (including lower weights and higher body image concerns) [44]. There is a paucity of effective pharmacological and/or psychological treatments for AN [65]. Reported rates of recovery vary and include 18% [56] to 52% at 6 year FU [7] to 60.3% at 13 year FU [20] and 62.8% at 22 year-FU [61]. Reported relapse rates in AN also vary, for example, 41.0% at 1 year post inpatient/day program treatment [35] to 30% at 22 year FU [61]. Average length of illness across the reviewed studies also varies from 6.5 years [56] to 14 years [41].

A variety of reported outcomes from treatment studies is likely due to the breadth of treatments under investigation, diverse study protocols and cohorts. For example, in a mixed cohort of female adult patients with AN and Atypical AN (A-AN), 33% were found to have made a full recovery at 3 year FU after treatment with cognitive behavioural therapy (CBT) [57], while 6.4% had a bad outcome and 6.4% a severe outcome. However, in a 5–10 year FU study of paediatric inpatients (mean age 12.5 years) approximately 41% had a good outcome, while 35% had intermediate and 24% poor outcome [66]. Multimodal treatment approaches including psychiatric, nutritional, and psychological rehabilitation have been found to be most efficacious for moderate to severe and enduring AN but noting a discrete rate of improvement [67].

Very few factors were able to predict outcomes in AN. Higher baseline BMI was consistently found to be the strongest predictor of recovery, and better outcomes were associated with shorter duration of illness [7, 55, 61, 66]. Earlier age of illness onset [59, 68, 69] and older age at presentation to treatment [30] were related to chronicity of illness and associated with poorer outcome.

There was a consensus across a variety of studies that engagement in binge/purge behaviours (Anorexia Nervosa Binge/Purge subtype; AN-BP) was associated with a poorer prognosis [20, 56, 70]. Similarly, individuals with severe and enduring AN restrictive sub-type (AN-R) are likely to have a better outcome than individuals with AN-BP. AN-BP was associated with a two-fold greater risk of relapse compared to AN-R [30, 35]. Some studies, however, were unable to find an association between AN subtype and outcome [55]. Other factors leading to poorer outcome and higher probability of relapse were combined ED presentations, such as combined AN/BN [35], higher shape concern [57], lower desired weight/BMI [44], more ED psychopathology at EOT, low or decreasing motivation to recover, and comorbid depression [35, 61].

Preliminary genetic work has found associations between a single nucleotide polymorphism (SNP) in a ghrelin production gene (TT genotype at 3056 T-C) and recovery from AN-R [71], and the S-allele of the 5-HTTLPR genotype increasing the risk susceptibility for both depressive comorbidity and diagnostic crossover at FU of AN patients [72]. These studies, however, need to be interpreted with caution as they were conducted over a decade ago and have not since been replicated. Research in eating disorder genetics is a rapidly emerging area with potential clinical implications for assessment and treatment.

#### Bulimia nervosa (BN)

Overall, studies pertaining to a diagnostic profile of BN report remission recovery rates of around 40–60%, depending on criteria and FU period, as detailed below. Less than 40% of people achieved full symptom abstinence [73] and relapse occurred in around 30% of individuals [61]. A meta-analysis of 79 case series studies reported rates of recovery for BN at 45.0% for full recovery and 27.0% for partial remission, with 23.0% experiencing a chronic course and high rates of treatment dropout [63]. At 11 year FU, 38.0% reported remission in BN patients, increasing to 42.0% at 21 year [45]. At 22 year FU, 68.2% with BN were reported to have recovered [41]. Higher frequency of both objective binge episodes and self-induced vomiting factors influencing poorer outcomes [44].

Considering impact of treatment, analysis of engagement in self-induced vomiting as a predictor for outcome indicated there were no differences between groups in treatment dropout or response to CBT among a sample of 152 patients with various types of EDs (AN-BP, BN, EDNOS) at EOT [74]. Meta-analysis of results from 45 RCTs on psychotherapies for BN found 35.4% of treatment completers achieved symptom abstinence [73] with other studies indicating similar rates of recovery (around 52–59% depending on DSM criteria) [7].

Studies delivering CBT or other behavioural therapies reported the best outcomes for BN [73]. Specifically, early treatment progression, elimination of dietary restraint and normalisation of eating behaviour resulted in more positive outcomes [22]. These findings are supported by results from a study comparing outcomes of CBT and integrative cognitive-affective therapy (ICAT) [75]. Additional moderating effects were shown at FU (but not EOT), with greater improvements for those with less baseline depression, higher stimulus seeking (the need for excitement and stimulation) and affective lability (the experience of overly intense and unstable emotions) in the ICAT-BN group and lower stimulus seeking in the Enhanced Cognitive Behavioural Therapy (CBT-E) group. Lower affective lability showed improvements in both treatment groups [75]. Such findings indicate personality factors may deem one treatment approach more suitable to an individual than another.

A review of 4 RCTs of psychotherapy treatments for BN in adolescents (including FBT and CBT) reported overall psychological symptom improvement by EOT predicting better outcomes at 12 months, which underscored the need for not only behavioural but psychological improvement during 6 month treatment [31]. Other factors leading to poorer outcomes included less engagement in treatment, higher drive for thinness, less global functioning, and older age at presentation [45]. More research is needed into consistent predictors, mediators and moderators focused on treatment engagement and outcomes [22].

While many studies combine findings for BN and BED, one study specifically considered different emotions associated with binge eating within the two diagnostic profiles [60]. At baseline, binge eating was associated with anger/frustration for BN and depression for BED. At FU, objective binge eating (OBE) reduction in frequency (a measure of recovery) was associated with lower impulsivity and shape concern for BN but lower emotional eating and depressive symptoms for BED. These differences may provide approaches for effective intervention targets for differing presentations; however, how these may play out within a transdiagnostic approach requires further enquiry.

#### Binge eating disorder (BED)

BED is estimated to affect 1.5% of women and 0.3% of men worldwide, with higher prevalence (but more transient) in adolescents. Most adults report longstanding symptoms, 94% lifetime mental health conditions and 23% had attempted suicide, yet only half were in recognised healthcare or treatment [76].

Compared with AN and BN, long-term outcomes, and treatment success for individuals with BED were more favourable. Meta-analysis of BED abstinence rates suggests available psychotherapy and behavioural interventions are more effective for this population [77]. Additionally, stimulant medication (i.e., Vyvanse) has been found to be particularly effective to reduce binge eating [see [78] for full review]. Results from a study of people who received 12 months of CBT for BED indicated high rates of treatment response and favourable outcomes, maintained to 4 year FU. Significant improvements were observed with binge abstinence increasing from 30.0% at post-treatment to 67.0% at FU [79]. A meta-analysis reviewing psychological or behavioural treatments found Interpersonal Therapy (IPT) to be the treatment producing the greatest abstinence rates [73]. In a comparative study of IPT and CBT, people receiving CBT experienced increased ED symptoms between treatment and 4 year FU, while those who received IPT improved during the same period. Rates of remission at 4 year FU were also higher for IPT (76.7%) versus CBT (52.0%) [80].

One study specifically explored clinical differences between ED subtypes with and without lifetime obesity over 10 years. Prevalence of lifetime obesity in ED was 28.8% (ranging from 5% in AN to 87% in BED), with a threefold increase in lifetime obesity observed over the previous decade. Observed with temporal changes, people with ED and obesity had higher levels of childhood and family obesity, older-age onset, longer ED duration, higher levels of ED (particularly BED and BN) and poorer general psychopathology than those who were not in the obese weight range [81], suggesting greater clinical severity and poorer outcomes for people of higher weight.

Comparison of 6 year treatment outcomes between CBT and Behavioural Weight Loss Treatment (BWLT) found CBT more effective at post-treatment but fading effectiveness over time, with remission rates for both interventions lower than other reported studies (37%) [82]. A meta-analytic evaluation of 114 published and unpublished psychological and medical treatments found psychological treatments, structured self-help, and a combination of the two were all effective at EOT and 12 month FU but noted a wide variation in study design and quality, and the need for longer term FU. Efficacy and FU data for pharmacological and surgical weight loss treatments were lacking [77].

Whilst high weight and associated interventions (such as bariatric surgery) can be associated with any ED, they are frequently studied in relation to BED. A significant proportion of individuals seeking bariatric surgery (up to 42%) displayed binge eating symptomatology [83], yet little is known about the effect of these interventions on ED psychopathology and whether this differs by type of intervention. A systematic review of 23 studies of changes in ED behaviour following three different bariatric procedures found no specific procedure led to long term changes in ED profiles or behaviours [84]; however, another study investigating the placement of an intragastric balloon in obese patients found post-surgical reductions in grazing behaviours, emotional eating and EDNOS scores [85]. Bariatric surgery in general is associated with a reduction in ED, binge eating and depressive symptoms [86].

Outcomes among patients receiving bariatric surgery with and without BED were assessed where weight loss was comparable between the groups at 1 year FU. However, compared with participants receiving a BWLT-based lifestyle modification intervention instead of surgery, bariatric surgery patients lost significantly less weight at a 10.3% difference between groups. There was no significant difference between lifestyle modification and surgery groups in BED remission rates [87]. These results indicate that BLWT-type interventions are more effective than surgery at promoting weight loss in individuals with BED over a 1 year FU period, and people with BED and higher BMI were able to maintain weight loss in response to psychotherapy (CBT) at up to 5 year FU [88]. In analysis of health-related quality of life (HRQoL) in people with BED who received various levels of CBT (therapist-led, therapist-assisted and self-help), evaluation indicated that all modalities resulted in improvements to HRQoL. Poorer outcomes were associated with obesity and ED symptom severity at presentation, stressing the importance of early detection and intervention measures [89]. Research into the role of CBT in strengthening the effect of bariatric surgery for obesity is ongoing but promising [90].

#### EDNOS, OSFED and UFED

Similarly to BED, a diagnosis of DSM-IV EDNOS (now OSFED) was associated with a more favourable outcome than AN or BN, including shorter time to remission. One study reported remission rates for both EDNOS and BED at 4 year FU of approximately 80% [21]. The researchers suggested that an ‘otherwise specified’ diagnostic group might be comprised of individuals transitioning into or out of an ED rather than between diagnostic categories; however, more work is needed in this area to fully understand this diagnostic profile. The reported recovery rate from EDNOS-A has been found to be much lower at 57% than for EDNOS-B at 80% (DSM-V). One factor suggested leading to poorer outcomes for EDNOS-A was a higher association with a co-occurring condition of major depression and/or dysthymia not found in other EDNOS subtypes [7]. Another study found purging occurred in 6.7% from total (cross-diagnostic) ED referrals, but this subtype did not have different post-treatment remission rates or completion rates compared to non-purging profiles [91], so results are mixed.

Acknowledging the scarcity of research within these diagnostic groups, remission rates for adolescents including those with a diagnosis of Other Specified Feeding or Eating Disorder (OSFED) and Unspecified Feeding or Eating Disorder (UFED) was reported to be 23% at 12 month FU in the one study reviewed, but no detail was provided on recovery rates by diagnosis [26]. No available evidence was identified specifically for the DSM-V disorders OSFED or UFED for adults.

#### Avoidant/Restrictive Food Intake Disorder (ARFID)

Research into outcomes for people with ARFID is lacking, with only three studies meeting criteria for the review [23–25]. While, like AN, recovery for people with ARFID is usually measured by weight gain targets, one of the three studies [63] identified by this review instead reported on outcomes in terms of meeting a psychiatric diagnosis, making comparison between the studies difficult.

In a cross-diagnostic inpatient study, individuals presenting with ARFID were younger, had fewer reported ED behaviours and co-occurring conditions, less weight loss and were less likely to be bradycardic than individuals presenting with AN [25]. Although both groups received similar caloric intakes, ARFID patients relied on more enteral nutrition and required longer hospitalisations but had higher rates of remission and fewer readmissions than AN patients at 12 months. This study highlights the need for further investigation into inpatient treatment optimisation for different diagnostic profiles.

People with ARFID who had achieved remission post-treatment were able to maintain remission until 2.5 year FU, with most continuing to use outpatient treatment services [23]. In a 1 year FU study assessing ARFID, 62.0% of patients had achieved remission as defined by weight recovery and no longer meeting DSM-V criteria [25]. In a study following children treated for ARFID to a mean FU of 16 years post-treatment (age at FU 16.5–29.9 years), 26.3% continued to meet diagnostic criteria for ARFID with no diagnostic crossover, suggesting symptom stability [24]. Rates of recovery for ARFID patients in this study were not significantly different to the comparison group who had childhood onset AN, indicating similar prognoses for these disorders. No predictors of outcome for patients with ARFID were identified by the articles reviewed [63].

#### Community outcomes

While most outcome studies derive from health care settings, two studies were identified exploring outcomes of ED within the community. The first reported the 8 year prevalence, incidence, impairment, duration, and trajectory of ED via annual diagnostic interview of 496 adolescent females. Controlling for age, lifetime prevalence was 7.0% for BN/subthreshold BN, 6.6% for BED/subthreshold BED, 3.4% for purging disorder, 3.6% for AN/atypical AN, and 11.5% for feeding and eating disorders not otherwise classified. Peak onset age across the ED diagnostic profiles was 16–20 years with an average episode duration ranging from 3 months for BN to a year for AN; researchers noted that these episodes were shorter than the average duration estimates reported in similar research and may be representative of the transient nature of illness rather than longer term prognosis. ED were associated with greater functional impairment, distress, suicidality, and increased use of mental health treatment [27].

A second study followed 70 young people (mean age of 14 years at study commencement) meeting DSM-IV criteria for a binge eating or purging ED and found 44% no longer met criteria at ages 17 or 20, while 25% still met criteria at age 20 (the latter individuals were more likely to have externalising behaviour problems and purging behaviour at age 17). Those who experienced a persistent ED were less likely to complete secondary education and report higher depressive and anxiety symptoms at age 20, indicating the ongoing impacts of ED on education and quality of life [92]. These studies provide information about the course and outcome of early onset ED at the population level with indicators of predictive and maintaining factors.

### Factors relating to outcomes

Several factors relating to outcomes have been studied across ED presentations and in specific diagnostic profiles. These include predictors of outcome, moderators or mediators of outcome, and illness reinforcers, considering age of presentation and duration of illness, ED symptomatology, presence of co-occurring medical and psychiatric conditions, and treatment characteristics.

#### Age of presentation

Age of presentation to treatment has been shown to have a significant impact on outcome in all diagnoses. One study considering ED in general (including AN, BN and EDNOS) showed presentation at mid-life drastically decreased chances of achieving a good outcome in response to treatment (“good” outcome defined as BMI ≥ 18.5, 3 month remission of symptoms and Eating Disorder Examination Questionnaire (EDE-Q) scores within or better than normal range). Six percent of mid-life (≥ 40 years) presentations achieved a good outcome post-treatment compared to 14% of young adults (18–39 years) and 28% of younger people (< 18 years) [28]. This finding has also been seen in research comparing 22 year outcomes of AN and BN [61].

People presenting in mid-life often have more complex medical and psychiatric profiles as well as life circumstances. They are also far more likely to have a sustained length of illness by the time of initial presentation: 27.8 years compared with 1.2 years for youths [28]. Longer duration of illness is associated with greater increase in self-reported clinical impairment [93]; however, illness duration does not necessarily influence treatment outcome, though wide variation in study protocol and quality limit the interpretability of these findings [37, 94]. The disparity in rates of favourable outcome between age groups highlights the importance of prevention, screening, awareness of ED in primary care settings and early intervention programs, as well as targeted programs for those presenting with more complex psychosocial and life challenges.

#### Clinical features and co-occurring conditions

A systematic review assessed the average duration of untreated illness duration in help-seeking populations at first contact to treatment services at 29.9 months for AN, 53.0 months for BN and 67.4 months for BED [69]. ED clinical factors significantly influence outcomes, with poorer prognosis in those with time of untreated illness, primary diagnosis of AN [95], lower BMI at presentation [93], and presence of binge/purge symptomatology [20, 56]. Certain ED behaviours and cognitions at intake predict better outcome such as lower rates of purging behaviour, higher rates of body image flexibility [96], and lower EDE-Q scores at baseline [97].

There is strong evidence for the presence of co-occurring medical and psychiatric conditions as a predictor of outcome in ED. At 22 year FU, the presence of co-occurring psychiatric conditions including Major Depressive Disorder (MDD) and Substance Use Disorder (SUD) were negatively correlated with recovery, with those who had recovered from an ED being 2.17 times less likely to have MDD and 5.33 times less likely to have SUD [98]. Co-occurring mood disorders consistently lead to poorer outcomes [47, 51, 55, 99] and greater chance of moving between ED diagnoses [7]. In one study, presence of a mood disorder was the strongest predictor of classification of AN-R (but not AN-BP) [61]. Comorbid personality disorder was found in several studies to be the most common predictor of poorer outcome in ED [20, 41, 44, 67].

In an adolescent sample, 39% of individuals with AN met criteria for at least one other psychiatric disorder and poorer prognosis was associated with co-occurring diagnoses of Obsessive Compulsive Disorder (OCD) and autistic traits [59]. In a large community childhood health longitudinal study, presence of any ED profile was predictive of later anxiety and mood disorders. AN was prospectively associated with long term low weight, while BN and BED with obesity, drug use and deliberate self-harm compared to age-matched children who did not have an ED profile [100].

Personality traits have also been found to be associated with poorer outcomes such as low persistence and harm avoidance in AN, lower self-directedness (BN) and reward dependence (BED) [41]. Higher perfectionism at intake predicted a lower likelihood of remission at 12 months in an adolescent sample [26], a finding consistent with previous research in adult cohorts [41].

Medical comorbidities such as malnutrition [72], concurrent type 1 diabetes [39, 42], bodily pain [55] and viral infections [72] have been identified as risk factors for poorer outcomes and increased rates of relapse. Other co-occurring factors associated with poorer outcomes for people with ED include anxiety [47, 56, 93], dissociative experiences [101], impulsivity [56], adjustment disorder [95], use of psychotropic medications [30], and autistic traits have been associated with greater use of ED treatment [102].

#### Psychosocial, environmental and health factors

A large United States community study found positive correlation between higher rates of smoking behaviour and ED in women [99]. The same study also reported birth-related outcomes in women with ED including having a later first birth, pregnancy health concerns, experience of miscarriage or abortion [99], and women with ED may have increased experience of adverse pregnancy and neonatal outcomes, and lower numbers of children [3]. For women with a history of ED, ED symptoms tend to alleviate during pregnancy; however, they commonly resurface during the postnatal period, and up to a third of women with ED report postnatal depression [103, 104].

Demographic factors leading to poorer prognosis include being male [72], of the LGBTQIA + community [105], being from a non-white ethnic background, low family education levels [99], lower socioeconomic status, living in a remote or rural area [72], poor employment and social adjustment [30], functional impairment [47], and having a family member with an ED [99]. Complicating prognosis are additional factors such as financial stress (individuals with ED face yearly health care costs 48% higher than the general population, while the presence of co-occurring psychiatric conditions is associated with 48% lower yearly earnings [3]. These financial challenges limit ability to access evidence-based treatments (especially in countries lacking in publicly funded health care) which may prolong illness.

There is strong evidence to suggest QoL is reduced in people with an ED [3, 106]. It is important to consider associations between QoL, ED symptomatology and treatment outcome. Evidence-based treatments have demonstrated positive effects on QoL in addition to reduction in ED symptomatology, for example, improvements in QoL and psychological functioning and well-being were seen in response to CBT in a cross-diagnostic sample [43]. However, a meta-analysis of ED outcome studies found that the QoL of recovered ED patients remained lower than in healthy populations, highlighting the importance of prevention efforts [107] and restoration of QoL in relapse prevention. These studies highlight the high public health and clinical burden of eating disorders and the need to consider co-occurring medical and psychiatric conditions during comprehensive assessment history-taking, treatment planning and provision.

#### Treatment factors

Early progression in treatment can provide indication of treatment outcomes. In an RCT comparing Family Based Treatment (FBT) and Adolescent Focused Therapy (AFT) for adolescents with AN, most people who achieved remission at 1 year FU maintained recovery to 4 years FU regardless of treatment arm with remission rates tended to remain stable after 1 year [108]. The First Episode Rapid Early Intervention for Eating Disorders (FREED) service model for young adults with AN reported significant and rapid clinical improvements in over 53.2% of people compared to 17.9% TAU and also reported more cost-effective treatment [109]. In a transdiagnostic study comparing inpatient vs outpatient settings, rapid response to treatment (defined here as a clinically meaningful reduction in disorder-specific symptoms within the first ten sessions) was the only outcome predictor accounting for 45.6% of variance in ED symptoms, suggesting future work should evaluate mediators and moderators of rapid response [37]. A systematic review of outcome predictors and mediators in response to CBT indicated that early behavioural and cognitive change was associated with positive outcomes across ED diagnoses [22]. Similarly, a recent systematic review and meta-analysis of 20 years of accumulated evidence concluded early response to treatment the most robust predictor of better treatment outcomes, however, only half of people investigated across numerous studies showed early change, and more research was needed to determine outcome predictors [110]. Ongoing assessment to identify individuals who do not show early response to treatment (defined by healthy weight and absence of ED behaviours at 12 month FU), as well as provision of targeted engagement approaches, may improve outcomes [47].

Due to the frequent need for medical stabilisation in the early and acute stages of AN, the role of hospitalisation needs to be considered in the evaluation of treatment outcomes. In a large patient cohort study (*n* = 7505) with 5 year FU, a clear trend was observed with the per-patient 5 year cumulated number of inpatient days decreasing by 6% per annum after adjustment for age at diagnosis, parental mental health, and household income. The number of hospital admissions decreased by 2% per year, although there was no change in outpatient visits [111]. Factors contributing to better outcomes were not identified in this study, but in other research, early change in %EBW and ED psychopathology in adolescent inpatients predicted later change in the same ED variables [18]. Another study showed longer first admission predicted increased use of the health system in young adults [112].

In a multicentre RCT there was no difference between higher or lower calorie refeeding on clinical remission or medical hospitalisation to 12 month FU [113]. A systematic scoping review of 49 studies found adolescent day programs (intensive treatment programmes that do not involve an overnight stay at the treatment facility) can be an effective alternative to inpatient hospitalisation or step up/down in treatment intensity and are generally associated with weight gain and improvements in ED and comorbid psychopathology [114]. Outcomes in the review were sustained from 3 months to 2 years from EOT; however, due to large variability in the content, structure and theoretical underpinnings of reviewed programs, findings should be interpreted with caution.

Difficulties with emotion regulation are also associated with poor outcome across diagnostic profiles. There is evidence to suggest emotion-focused treatment is beneficial both to emotional functioning and mood as well as ED severity for people with elevated emotion regulation issues at baseline with positive effects lasting up to 5 years FU [115].

#### Self-esteem, self-compassion, and motivation

There is little conclusive evidence regarding predictors of poor response to evidence-based treatments [22, 58]; however, low self-esteem has been implicated across all ED diagnoses [98, 101], particularly AN [55]. A meta-analysis exploring the role of self-esteem on treatment outcomes indicated that while self-esteem did not predict remission or long-term weight related outcomes, it did mediate progression during inpatient treatment (greater increase in self-esteem during inpatient treatment was associated with higher remission and lower relapse rates at FU) [116]. Relatedly, high fear of self-compassion was associated with greater severity of ED symptoms in individuals with an active ED, suggesting that a fearful unwillingness to become more self-compassionate, rather than the absence of self-compassion, may lead to more detrimental outcomes [117].

Greater pre-treatment motivation has also been associated with ED symptom improvement and management of co-occurring anxiety and depression, in a systematic review and meta-analysis of 42 longitudinal studies [118]. Therapeutic interventions that include enhancement of motivation, self-esteem and self-compassion have been shown across studies to improve treatment outcomes across diagnostic profiles [117].

### Relapse prevention programs

Whilst the role of treatment is crucial in the alleviation of symptoms and restoration of wellbeing, active provision of evidence-based post-treatment recovery care may be an important determining factor in relapse prevention. Research suggests the period in which individuals are at greatest risk of relapse is between four and nine-months following discharge [35], with between 31 and 41% relapsing at one to two years post-discharge [62].

To reduce readmission among a group of females receiving inpatient treatment for AN at an Australian specialist child and adolescent ED service, a 10 week transition ‘day’ program was developed and evaluated. The delivered program allowed for a ‘step down’ option and was found to have significant benefit for participants, who achieved an average weight gain of over 1 BMI point and decreased ED symptomatology at six-month FU [65]. Promising findings were also seen in a 6-session post-(inpatient and/or outpatient) treatment relapse prevention program designed by clinicians, parents, and patients in the Netherlands, which included a take-home workbook and appointments up to 18 months (frequency dependent on patient progress). Evaluated with young people with AN-R and AN-BP, 70% maintained post-discharge recovery to the end of the study period [36]. Such programs were evaluated in the context of a comprehensive specialist service with no control group comparison to measure the impact of the specific intervention, and there was no FU assessment following conclusion of the intervention to assess maintenance. Although more work is needed, these studies indicate the value of targeted relapse prevention programs.

#### Online relapse prevention programs

There is emerging evidence to support the safety and efficacy of internet-based relapse prevention programs aimed at preventing readmission to intensive ED treatment following discharge. These programs have the potential to be widely disseminated to individuals who may otherwise disengage from ongoing support due to access issues (e.g., living in an underserviced area, financial burden) or personal reasons such as stigma or shame [119, 120].

A 9-session (1/month) CBT-based online relapse prevention program for women with AN discharged from inpatient treatment (baseline BMI *x̄* = 17.7) found participants who completed the program had significant gains in BMI at end of program (*x̄* = 19.1) while the treatment as usual (TAU) control group did not (*x̄* = 17.7). Of note, participants who were 1–2 sessions short of completing the program maintained a higher BMI (*x̄* = 18.0) than the TAU group, whereas participants with less than 50% completion had a significantly lower BMI than any group including TAU (*x̄* = 17.0) [121]. A similar CBT-based online program targeted toward women discharged from inpatient treatment for BN found that the intervention group reported 46.0% fewer vomiting episodes compared to TAU, with some improvement in symptom abstinence (intervention group: 21.4%, TAU control = 18.9%), although this finding was not statistically significant [122].

In Hungary, an internet-based aftercare support program for individuals who had received inpatient or outpatient treatment for BN or related EDNOS in the 12 months prior to the study included information and support offered via 30 min chat sessions with peers and clinicians. Results showed 40.6% of the intervention group reported improvement compared to TAU waitlist controls (24.4%), although this difference was not statistically significant. The study noted that, although on the waitlist for the internet-based aftercare support program, the TAU group could still access additional treatment if so required. Evaluation findings report the program was feasible and well accepted [123].

Text messaging-based interventions have also been trialled to maintain engagement post-treatment, whereby participants send regular symptom reports to the clinical team with feedback provided. A 12 week ‘mobile therapy’ study with a group of women exiting CBT treatment for BN resulted in significant improvement in binge/purge frequency, ED and depressive symptoms from baseline to FU, with high rates of protocol adherence (87.0%), although there was no control group comparison [124]. Further evidence was provided in a 16 week weekly symptom report study of women with BN following inpatient discharge, with a significantly larger proportion of the intervention group achieving remission (51%) compared with TAU (36%) at 8 months FU. There was no significant difference between groups in terms of outpatient service use [125]. Results from these studies conflict with evidence from a systematic review of 15 studies, which was unable to support the effectiveness of text messaging-based programs for people with ED as either a sole or adjunctive component of the intervention [126]; however, this review noted the lack of a common evaluation framework making comparison difficult.

### Mortality

Despite advances in awareness and treatment, ED, particularly AN, continue to be associated with increased risk of mortality [4]. Studies identified that focus on the assessment of ED mortality, as well as data from the *Global Burden of Disease Study 2016* are discussed in this section. Importantly, there are several different metrics used to report mortality. These include the *Standardised Mortality Ratio* (SMR), or the number of observed deaths in a cohort versus the number of expected deaths in a reference population (where a rate greater than one is interpreted as excess mortality); *Weighted Mortality Ratio* (WMR), or the weighted average of age-specific mortality rates per 100,000 persons; *Crude Mortality Rate (CMR)*, or the number of deaths in a given period divided by the population exposed to risk of death in that period; and *Years of Life Lost* (YLL), a summary measure of premature mortality calculated by subtracting the age at death from the standard life expectancy in a reference population.

#### Standardised, weighted, and crude mortality

AN is consistently described as having the highest mortality rate of the ED, but actual rate difference varies between studies. A summary of Standardised Mortality Ratios across studies is presented in Table 3. SMRs from a meta-analysis suggest that measured mortality of AN is approximately three times as high as for other ED diagnoses, and in a UK study of ED patients (*n* = 1892) accessing services between 1992 and 2004, the SMR for AN was almost five times higher than other ED [127]. This is consistent with other research (a meta-analysis summarising 41 studies) reporting people with AN were 5.2 [3.7–7.5] times more likely to die prematurely from any cause [128]. A longitudinal study (*n* = 246) found SMR of AN to be only twice as high compared to BN, but still 6.5 times the rate expected in the general population [49].

**Table 3:** Standardised mortality rate by eating disorder diagnosis across included studies

	ED	AN	BN	BED	EDNOS/OSFED	*n*
Ward et al. [129]						
Female		6.3	2.3	2.2	2.1	100,000
Male		6.7	2.3	2.0	2.1	
Nielsen et al. [130]						
Female		3.4	1.8	2.3	4.1/3.7	21,325
Male		3.6	2.6	3.1	4.7/4.3	1308
Iwajomo et al. [131]						
Female	4.6					17,108
Male	7.2					1933
All	5.1					19,041
Arcelus et al. [4]						
All		5.9	1.9		1.9	17,272
Hoang et al. [132]						
Female	2.2	5.0	4.8		1.6	13,449
Male	1.8	2.7	2.2		1.6	1646
All	1.8	3.6	3.2		1.3	15,095
Keshaviah et al. [128]						
All		6.5				12,071
Fichter et al. [2]						
Female		5.3	1.6		1.9	5296
Male		4.9	1.4		1.9	188
Fichter and Quadflieg [133]						
All		5.4	1.5	1.5	2.4	5839
Suokas et al. [134]*						
Female	2.7					2337
Male	9.1					113
All	2.93	3.5	2.7	3.3		2450
Crow et al. [135]						
All		1.7	1.6		1.8	1885
Button et al. [127]						
Female		9.8	1.6		2.5	1803
Castellini et al. [136]						
All	1.19	2.49	2.07	1.01		1277
Huas et al. [68]						
Female		10.6				601
Rigaud et al. [20]						
Female		1.3				464
Male		0				22
All		1.2				484
Guinhut et al. [137]						
Female		15.7				363
Male		22.4				21
All		15.9				384
Quadflieg et al. [138]						
Male		5.9	1.9		3.4	338
Franko et al. [49]						
All		4.4	2.3			246
Rosling et al. [139]						
All		11.7	4.0			201

*Mortality rate is reported by per 1000 person-year

Some studies did not report higher SMR for AN compared to other ED, however, methodological differences need to be considered. For example, some studies reported comparable SMR for AN to other ED, but subthreshold AN cases were included (previously catagorised as EDNOS) which may have reduced the calculated AN SMR [104, 108]. In a British study using English National Hospital Episodes Statistics (2001–2009) comparing AN and BN, little difference in SMRs was reported [132]. The diagnosis of BN was less likely than other diagnosis to be recorded as the primary diagnosis and may not have been representative.

In a 22 year trial FU of a large sample of inpatients treated for BN, 2.4% had died [45]; the CMR for BN was 0.32% [63] and in severely malnourished patients, the crude mortality rate rose to 11.5% with SMR 15.9 [CI 95% (11.6–21.4)], just over 5 years post-treatment [137]. WMR has been found to be 5.1 for AN, 1.7 for BN, and 3.3 for EDNOS. SMRs were 5.86 for AN, 1.93 for BN, 1.92 for EDNOS [4] and 1.5–1.8 for BED [76].

Mortality rates in AN were highest during the first year after admission to treatment, while in BN it is in the first two years [134], with a higher risk in adolescence [140]. In AN, peak age of risk of death has been reported to be 15 years of age, BN 22 years and EDNOS 18–22 years [141]. Substance use disorders (including alcohol and/or cannabis) increased mortality in people with eating disorders across the diagnostic profiles [142].

In ED, peak age of risk for males may be earlier than females [141]. SMRs are higher for males (SMR = 7.24; 95% CI 6.58–7.96) relative to females (SMR = 4.59; 95% CI 4.34–4.85) overall, and in all age groups [131]. This may be due to the lower likelihood of males to self-identify or be identified with ED resulting in treatment delays and higher severity of illness when finally seeking help [131]. In mortality research conducted with a male-only sample, similarly high SMRs for males with BN and particularly AN as in majority female samples [2] were reported; however, mortality rates of EDNOS in males were considerably higher than those reported in female-dominant or female-only samples. Moreover, a case-controlled study found there was a sex difference across all diagnostic categories in CMR, with male to female being 15–5% in AN, 8–3% in BN, and 4–3% in EDNOS, but there were no significant sex differences in SMR for any diagnostic group, with males showing a shorter survival time after onset [2]. Researchers have suggested that increased mortality in males could be due to several factors, including reluctance to seek treatment and current treatment approaches being less effective in males [138]. Further research in males with ED is required to better understand the impact and response in male patients. Regardless of the mortality metric used, these studies indicate the vital importance of considering elevated mortality risk across the range of ED diagnoses.

#### Years of life lost/years lived with disability

The Global Burden of Disease Study 2016 reported that YLL due to premature death attributable to AN was 0.4 per 100,000. No YLL were attributed to BN; however, cause-specific mortality (CSM)—where each death is attributed to a single underlying cause—was, per thousand, 0.5 for AN (with a 2.9% increase from 1980 to 2016) and 0.1 for BN (21.8% increase from 1980 to 2016) [143]. The 2019 extension advocated for the inclusion of BED and OSFED in the Global Burden of Disease Study, previously excluded, as both diagnostic groups accounted for the majority of global ED cases and accounted for an unrepresented 41.9 million people living with ED [144].

Estimates are that over 3.3 million healthy life years are lost per year worldwide due to eating disorders. Years lived with a disability (YLDs) have increased from 2007 to 2017 for both AN (6.2% increase) and BN (10.3%), a higher rate than other mental disorders (− 0.1%). ED outcomes include reduced self-reported quality of life and estimated health care costs at 48% higher than for the general population [3].

#### Risk factors

Little is known about specific risk factors for mortality, although some variables have been reported in the literature. People who receive inpatient treatment for AN have more than five to seven times mortality risk when matched to age and gender and compared to other ED diagnoses [3, 131, 133]. For individuals receiving AN or BN treatment in outpatient settings, the risk is still twice that of controls [3]. Older age of presentation is a significant risk; adult presentations are associated with much higher mortality rates than adolescent presentations likely due to longer duration of illness at presentation, higher rates of medical and psychiatric complications and less engagement in treatment [4, 28, 68, 137, 139]. Higher mortality rates (especially in AN) are associated with lower BMI, longer duration of illness at service presentation [4, 49, 68, 137, 139], diuretic use [68], and occurrence of an in-hospital suicide attempt [68, 137]. Certain treatment factors may be associated with higher risk of mortality, including transfer to medical intensive care unit, discharge against medical advice, and shorter hospital stays [137]. Other factors associated with increased risk of mortality include poor psychosocial functioning, substance use [28, 49] and absence of family ED history [28].

#### Cause of death

Results from a large prospective 20 year (1985–2005) longitudinal study of individuals admitted to inpatient services in Germany (*n* = 5839) showed people with AN were likely to die from health issues caused by their disorder, most commonly circulatory failure, cachexia, and multiple organ failure [133]. Other studies have identified somatic risk factors including anaemia, dysnatremia, infection, cardiac complications and haematological comorbidities [137]. A 2021 study reported rates of medical complications for severe AN, which included anaemia (79%), neutropenia (53.9%), hypertransaminasemia (53.7%), osteoporosis (46.3%), hypokalemia (39.5%), hypophosphatemia (26%), hypoglycaemia (13.8%), infectious complications (24.3%), cardiac dysfunction (7.1%), and proven gelatinous bone marrow transformation (6.5%). Five (1.4%) of the patients in this study died of the following causes: septic shock of pulmonary origin (*n* = 1), septic shock of urinary origin (*n* = 1) and suicide (*n* = 3) [145].

Suicide is the most common non-natural cause of death in people with AN, BN, BED and EDNOS [133]. High rates of suicidality were reported in a meta-analysis of 36 studies published between 1966 and 2010 with data showing one in five individuals who died from an ED did so by suicide [4]. Risk of suicide may be particularly elevated in AN [Hazard Ratio (HR) 5.07; 95% CI 1.37–18.84] and BN (HR 6.07; 95% CI 2.47–14.89) even when specialised treatments are available [134]: people with AN are 18.1 [11.5–28.7] times more likely to die by suicide than 15–34 year old females in the general population [128]. This is supported by results from a meta-review exploring risk of all-cause and suicide across major mental disorders. 1.7 million patients and over a quarter of a million deaths were examined, finding all mental health disorders had an increased mortality rate to the general population; however, substance use and AN were the highest, translating into 10–20 year reductions in life expectancy, with borderline personality disorder, AN, depression and bipolar disorder having the highest suicide risk [146].

## Discussion

This rapid review, which synthesised the available literature on ED remission, relapse and recovery rates including associated moderating and mediating variables such as psychosocial and treatment characteristics, highlighted significant challenges of synthesising outcome literature. This includes a wide variety of ways in which key outcomes ‘remission’, ‘relapse’ and ‘recovery’ are not only defined but also how they are measured and analysed. There is no consensus among clinical or research communities on these definitions for any of the ED diagnoses [30, 31, 94]; thus, comparison between studies is challenging.

As EDs have amongst the highest rates of mortality of the mental health disorders, including one in five deaths caused by suicide, research into preventable causes of death, mitigatable risk, prevention and treatment efficacy is of paramount importance. It is noteworthy that current reported YLL and YLD for ED are likely an underestimate due to lack of robust epidemiological data, methodological limitations of burden of disease studies, absence of the illness group from national surveys and underreporting of mortality [147].

‘Relapse’ is typically defined by a return of symptoms after a period of reduced symptomatology; however, reviewed studies report a variety of methods to measure this, including multidisciplinary healthcare team assessment, scores on standardised psychological and behavioural interviews or questionnaires, weight criteria (including BMI or %EBW), reported eating disorder behaviours, meeting DSM (IV or V) diagnostic criteria, or a combination of the above. More difficult is determining if there is a difference between ‘remission’ and ‘recovery’, with remission usually determined by an absence of diagnostic symptomatology (again, characterised by a variety of methods), and recovery an improvement in overall functioning. Many studies report remission and recovery interchangeably, and very few incorporate returns to psychosocial functioning and QoL post alleviation of symptoms [29]. More standardised definitions may progress research [148] by allowing direct comparison between outcome studies, improving the ability of future investigations to predict and report relapse versus recovery rates and to comprehensively evaluate intervention and relapse prevention approaches.

An additional challenge across studies is a highly variable period between initial assessment or baseline and the time at which ‘outcome’ is assessed—ranging from as little as one week up to 25 years. As rates of relapse increase with illness progression, relatively short FU periods may compromise the understanding of true long-term outcomes. Longer-term FU studies are crucial to understand optimised models of care for sustained recovery and wellbeing.

Along with illness progression over time in individuals, the shift of diagnostic profiles among the individual may differ the definition of relapse or remission and thus impacts on outcome measures. Most research protocols adopt a firm inclusion/exclusion criterion, focusing on specific diagnostic profiles; however, findings from this review suggest considering a transdiagnostic approach in outcomes research which may better reflect the potentially transient nature of ED symptomatology [44]. This may have implications for diagnoses such as OSFED, potentially a transient category [21], rather than categorisation in or out of full ED diagnostic syndromes. Identification and consideration of transdiagnostic profiles, combined ED presentations and co-occurring mental health conditions should be considered in the long-term management and monitoring of individuals.

Studies within this review reported on cohorts of individuals with a formal diagnosis and research conducted within treatment settings. However, previous research has suggested that incidence rates within the community are considerable, and yet help-seeking of any type for a problem related to ED symptoms is uncommon, ranging between 22 and 40% [106] and there can be a significant time delay from first symptom experience [69]. A recent large community survey of the impact of COVID-19 on people with ED reported up to 70% of people who experienced ED symptoms were not in treatment [149] suggesting a significant proportion of people with an ED are not captured within this outcome review. Outcomes for this population are largely unknown [150] but preliminary research suggests they may be less favourable [151, 152].

Improved QoL has been shown to be a significant predictor of positive outcome and is an opportunity for broader scope interventions for people with ED [107], and yet consistent and more wholistic markers of life quality are rarely integrated into research or clinical decision making [153, 154]. It is also noted that outcome determinants in the reviewed studies are predominantly biometric (e.g., weight) and ED symptom related, whereas qualitative lived experience evidence suggests a broader range of person-centred metrics should be used to measure outcome. These include supportive relationships (e.g., receiving support, advice and encouragement from others, including family, friends, and/or professional carers), sense of hope, identity, meaning and purpose, feelings of empowerment and self-compassion [155]. Involvement of those to whom the work pertains (i.e., individuals with lived experience) is essential in future outcomes research to add richness and utility to theoretical frameworks, methodological approaches and conclusions [156].

### Key findings

ED frequently take a chronic course, with less than half of individuals achieving recovery at long-term FU [41, 44, 52]. Between 30 and 41% of people will relapse within two years of receiving treatment [35, 61], and between 20 and 61% will experience more than one type of eating disorder [7, 63, 64]. As with much of the extant ED literature, most outcome research has been conducted in AN. Restrictive ED are consistently associated with the poorest prognosis. This review identified recovery rates in the range of 18–60% for AN and an average length of illness of between 6.5 and 14 years [41, 56]. Binge/purge symptomatology within AN is associated with worse outcome [20, 56]. Recovery rates for BN are slightly more optimistic at 35–59% [7, 45, 63, 157], and similarly for BED at 37–77% [79, 80, 82]. There is limited data available on outcomes in ARFID, OSFED, and UFED.

Factors associated with a more positive long-term outcome include lower age of presentation [28, 61], shorter duration of illness at first presentation [69, 93, 94], higher pre-treatment motivation to recover [116], and demonstrated early response to treatment [18, 75, 110, 112]. Factors associated with poorer outcome are lower BMI at presentation [93], presence of binge/purge symptomatology [20, 30, 44, 56], and presence of comorbid psychiatric condition/s such as depression, anxiety, or personality disorder [44, 47, 51, 55, 67, 98, 99]. Males, LGBTQIA + community [104, 105], neurodiversity [102], individuals from non-white/ethnic backgrounds, and those from lower socioeconomic brackets or rural/remote communities are also more likely to experience a poor outcome [18, 72, 76, 77].

Relapse following ED treatment is common [11, 35, 36, 62, 148] and is most likely to occur 4–9 months post discharge [35]. Up to 41% of individuals will relapse by the second-year post-discharge [62]. Aftercare relapse prevention programs, including online and face-to-face initiatives such as text-message based interventions, daily feedback to clinicians and intensive day programs have been shown to increase chance of maintaining recovery [121, 123–125]. The implementation of such programs may be key to improving long-term recovery rates particularly for those individuals who may otherwise disengage from treatment for access reasons (such as living in an underserviced area) or because of the stigma of engaging with mental health care [119, 120]. There is emerging evidence in the effectiveness of online intervention for preventing relapse and promoting treatment gains when individuals are motivated to change; however, evidence is not conclusive potentially due to the high variability of the interventions and evaluations of such programs.

ED are associated with unacceptably high mortality rates, and particularly high risk of suicide [128, 133]. Of the ED, AN carries the highest mortality risk [49, 127, 128]. Standardised mortality ratios (SMRs) identified by this review ranged between 1.2 and 15.9 for AN; 1.4 and 4.8 for BN; 1.01 and 3.3 for BED; and 1.3 to 4.7 for EDNOS/OSFED [2, 4, 20, 121, 127, 128, 132, 134, 135, 137, 139]. Factors associated with increased risk of mortality include having received inpatient treatment [3, 131, 133], longer duration of untreated illness [4, 28, 68, 68, 137, 139] and lower BMI at presentation [4, 49, 68, 137, 139]. Males are at higher risk of death than females [2].

### Strengths and limitations

This rapid review has several strengths inherent to the methodological approach of the series, conducted to inform the Australian Eating Disorders Research and Translation Strategy 2021–2031 [1]. The RR process broadly assessed all available high-level evidence peer-reviewed literature swiftly [24], included all diagnostic categories covering transdiagnostic continuums, considered the full demographic range available and reported a variety of methodological designs including clinical trials (across a variety of settings), systematic reviews, meta-analyses, and population-level research. It aimed to provide the most comprehensive and current review possible with coordination of complex findings into a more cohesive structure. It was noted where applicable the limitations of conclusions drawn from this review, such as the widely disparate definitions and measurements for key outcome data (i.e., remission, relapse, and recovery rates), crossover from DSM-IV to DSM-V criteria (due to timeframe of search), vastly different periods of follow up impacting findings, and conflicting evidence. As with the series of rapid reviews, the inclusion criteria of evidence may have potentially excluded relevant evidence, and it is noted that evidence is always emerging.

## Conclusion

This RR of outcomes in ED identified several gaps in current knowledge and provides direction for future strategic research directives, specifically, defining the key outcomes of remission, recovery, and relapse, with consensus of determinants and inclusion of broader QoL measures and lived experience. Identifying and refining risk factors, mediating and moderating factors that may influence outcomes is ongoing, with longer-term FU research needed to track remission versus relapse, diagnostic crossover and optimisation of treatment engagement and recovery. Regarding mortality literature, this review noted considerable gaps [146], with variety reporting methods, a paucity of research between population level reporting and small hospital outcome studies, and minimal investigation into life circumstances relating to death, especially as many of these deaths may be preventable. With low rates of remission despite evidence-based care and high risk of mortality, especially for AN, it is strongly recommended that focused, long-term follow-up research is prioritised for people with ED.

## Supplementary Information

**Additional file 1: Fig. S1.** PRISMA flow diagram.

**Additional file 2: Table S1.** Studies included in the Rapid Review.

## Data Availability

Not applicable—all citations provided.

## Abbreviations

A-AN: Atypical anorexia nervosa
AFT: Adolescent focused therapy
AN: Anorexia nervosa
AN-BP: Anorexia nervosa binge/purge subtype
AN-R: Anorexia nervosa restricting subtype
ARFID: Avoidant restrictive food intake disorder
BED: Binge eating disorder
BMI: Body mass index
BN: Bulimia nervosa
BWLT: Behavioural weight loss therapy
CBT: Cognitive behaviour therapy
CBT-E: Enhanced cognitive behavioural therapy
CMR: Crude mortality rate
DSM: Diagnostic and statistical manual of mental disorders
EBW: Expected body weight
ED: Eating disorders
EDE-Q: Eating disorder examination questionnaire
EDNOS: Eating disorder not otherwise specified
EDNOS-A: Eating disorder not otherwise specified-anorectic type
EDNOS-B: Eating disorder not otherwise specified-bulimic type
EOT: End of treatment
FBT: Family-based therapy
FU: Follow up
HMA: Healthcare management advisors
HRQoL: Health related quality of life
ICAT: Integrative cognitive-affective therapy
IOI: InsideOut Institute
IPT: Interpersonal therapy
MDD: Major depressive disorder
OBE: Objective binge eating
OCD: Obsessive compulsive disorder
OSFED: Other specified feeding or eating disorder
QoL: Quality of life
RCT: Randomised controlled trial
RR: National eating disorder research & translation strategy rapid review
SMR: Standardised mortality ratio
SUD: Substance use disorder
TAU: Treatment as usual
UFED: Unspecified feeding or eating disorder
WMR: Weighted mortality ratio
YLL: Years of life lost
YLD: Years lived with a disability
