# Supplementary material for: Eating disorder outcomes: findings from a rapid review of over a decade of research
Source: J Eat Disord. 2023 May 30;11:85. doi: 10.1186/s40337-023-00801-3 (PMC10228434; doi:10.1186/s40337-023-00801-3)
Supplement: Supplementary file 1 — Additional file 1: Fig. S1. PRISMA flow diagram. [file 40337_2023_801_MOESM1_ESM.docx]

Expert authors & other sources related to the current topic

(n=10)

articles included in current Rapid Review topic

(n=116)

Articles included in current Rapid Review topic

(n=106)

Articles identified through database searching

(n=17,757)

Articles identified through links and reference lists

(n=36)

Articles after duplicates removed

(n=9,260)

Articles screened through assessment of title/abstract

(n=9,260)

Excluded

(n=7,292)

Excluded

(n=660)

Full text articles assessed for eligibility

(n=1,968)

Expert research collaborative requested articles

(n=12)

Articles included in original Rapid Review

(n=1,320)

Identification

Screening

Eligibility

Included

**Figure 1.** PRISMA flow diagram
