# Supplementary material for: Eating disorder outcomes: findings from a rapid review of over a decade of research
Source: J Eat Disord. 2023 May 30;11:85. doi: 10.1186/s40337-023-00801-3 (PMC10228434; doi:10.1186/s40337-023-00801-3)
Supplement: Supplementary file 2 — Additional file 2: Table S1. Studies included in the Rapid Review. [file 40337_2023_801_MOESM2_ESM.docx]

**Table 1.** Studies included in the Rapid Review (Outcomes)

| **Author, Year** | **Country** | **Population** | **N**  **studies** | **N participants** | **Aim** | **Design** | **Outcome Measure** | **Ref. No.** |
| --- | --- | --- | --- | --- | --- | --- | --- | --- |
| Remission, recovery, and relapse, including outcome predictors, mediators, and moderators | | | | | | | | |
| Accurso et al., 2016 | USA | Outpatient (Adult, Both Sexes) |  | 80 | To examine predictors and moderators (incl. depression, anxiety, emotion dysregulation, affective lability, stimulus seeking, intimacy problems, actual-ideal self-discrepancy, actual-ought self-discrepancy, self-blame, and self-control) of outcome in two treatments (ICAT-BN or CBT-E) for bulimia nervosa (BN). | RCT | Eating disorder psychopathology at end of treatment and four-month follow-up | 75 |
| Agras et al., 2009 | USA | Mixed: community and specialty clinics (Adult, Both Sexes) |  | 385 | To examine the course of Eating Disorder NOS (EDNOS) compared with anorexia nervosa (AN), bulimia nervosa (BN), and binge eating disorder (BED). | Longitudinal (<5yr) | Relapse and remission; time to remission in AN, BN, BED and EDNOS. | 21 |
| Allen et al., 2013 | Australia | Community (Young people aged <20, Both Sexes) |  | 1383 | To describe the course of early onset eating disorders in a population-based sample followed from 14 to 20 years; identify variables that could account for the persistence of eating disorders from 14 to 20 years; and describe outcome of early onset eating disorders with reference to general and psychological functioning at age 20. | Longitudinal (>10yr) | ED psychopathology, anxiety and depressive disorders, behavioural problems. | 92 |
| Amianto et al. 2017 | Italy | Outpatient (Adult, Females) |  | 59 | To report on the 8 year follow up of a sample of AN patients treated with an integrated treatment model. | Longitudinal (>5yr) | BMI, ED psychopathology, personality features and comorbid psychopathology at follow-up. | 68 |
| Ando et al., 2010 | Japan | Community (Adult, Females) |  | 165 | To examine whether the ghrelin gene 3056 T→C SNP was related to changes from AN-R to other subtypes of eating disorders. | Cross-sectional (Correlational) | The 3056 T-C SNP of the ghrelin gene | 71 |
| Austin et al., 2021 | Worldwide | Community (Adult, Both Sexes) | 14 |  | To evaluate the average duration of untreated ED (DUED) in help-seeking populations at the time of first ED treatment and investigate the association between DUED and symptom severity/clinical outcomes | Review (systematic) | Clinical outcomes of ED, length and progression of ED or DUED | 70 |
| Austin et al., 2022 | UK | Community (Adult, Both Sexes) |  | 278 | To examine ED and other clinical outcomes over time within the FREED group and within clinical subgroups, to compare change in BMI for FREED (First Episode Rapid Early Intervention for ED) and TAU (Treatment as usual) participants with AN, and to compare service use between FREED and TAU patients. | Quasi-experimental | Recovery rate, EDE-Q, CORE-10, psychosocial impairment due to ED, depression, anxiety and stress (DASS-21), BMI | 111 |
| Bardone-Cone et al., 2010 | USA | Mixed: clinical and community (Aged >16, Females) |  | 155 | To operationalise a way of defining eating disorder recovery based on theory and prior research; and to test the validity of this definition by examining how a fully recovered group compare to healthy controls, a partially recovered eating disorder group, and an active eating disorder group using eating disorder assessments not used in defining recovery. | Cross-sectional (Correlational) | Psychosocial functioning and psychiatric comorbidity | 29 |
| Baudinet et al., 2021 | Worldwide | N/A | 49 |  | To review existing literature on adolescent eating disorder DP treatment models and outcomes. | Review (Systematic) | Physical Health, Eating Disorder and Comorbid Psychopathology, Psycho-Social Functioning, Quality of Life and Motivation, Family Factors and Outcome | 116 |
| Berends et al., 2018 | The Netherlands | Outpatient (Adult, Females) | 16 |  | To systematically review the existing literature on relapse in anorexia nervosa. | Longitudinal (<5yr) | Rate of relapse, timing of relapse, factors associated with relapse | 36 |
| Bluett et al., 2016 | USA | Inpatient (Adult, Females) |  | 113 | To test whether pre-treatment levels of psychological flexibility would longitudinally predict quality of life and eating disorder risk in patients at a residential treatment facility for eating disorders. | Repeated Measure (without follow-up) | Body image acceptance and action; ED psychopathology using EDI-3;and EDQoL at baseline and EOT. | 96 |
| Brown et al., 2020 | USA | Outpatient (Young People, Both Sexes) |  | 99 | To examine whether early change in weight and ED psychopathology predicts outcome for adolescents with anorexia nervosa (AN) in a partial hospitalization program. | Repeated Measure (with follow-up) | Remission: BMI percentile, ED psychopathology using EDE-Q | 18 |
| Bryson et al., 2018 | USA | Outpatient (Young People, Both Sexes) |  | 137 | To assess long-term outcomes of patients with avoidant/restrictive food intake disorder (ARFID) treated in a partial hospitalization program (PHP) for eating disorders (ED). | Longitudinal (<5yr) | BMI, ED psychopathology using ChEAT at follow-up. | 23 |
| Bye et al., 2021 | Worldwide | N/A | 15 |  | To provide an up-to-date overview of the impact of maternal ED on pregnancy and the postnatal period, and discuss clinical implications for identification and management of maternal ED. | Review | Impact of maternal ED in pregnancy and postpartum; remission; psychiatric comorbidities during perinatal period | 105 |
| Calugi et al., 2016 | Italy | Inpatient (Adult, Females) |  | 108 | To compare the long-term effects of a residential cognitive-behavioural treatment (CBT) for weight loss in severely obese patients with and without binge-eating disorder (BED). | Longitudinal (<5yr) | Weight loss, ED psychopathology, general psychopathology, and quality of life at follow-up. | 88 |
| Carter et al., 2012 | Canada | Inpatient (Adult, Both Sexes) |  | 100 | To identify predictors of relapse in adult AN using a prospective, longitudinal design. | Longitudinal (<5yr) | Incidence of relapse at follow-up and predictor variables (demographic and clinical features, behavioral and psychological changes during treatment, residual psychopathology at post-treatment, and motivation to recover) | 35 |
| Castellini et al., 2012 | Italy | Outpatient (Adult, Both Sexes) |  | 218 | To evaluate the relationships between psychopathological variables, and objective and subjective binge eating episodes in BN and BED at 3-year follow-up. | Longitudinal (<5yr) | OBEs and SBEs, ED psychopathology, anxiety and depressive symptomatology, body image concern and impulsivity at follow-up. | 60 |
| Castellini et al., 2012 | Italy | Outpatient (Adult, Both Sexes) |  | 201 | The aim of the present naturalistic study was to detect the possible relationships between the 5-HTTLPR polymorphism, the specific and general eating disorder psychopathology, and the response to treatment in AN and BN patients. | Longitudinal (>5yr) | Recovery (ED psychopathology & BMI) and diagnostic crossover at 6 year follow-up | 72 |
| Castellini et al., 2011 | Italy | Outpatient (Adult, Both Sexes) |  | 793 | To evaluate in a 6-year follow-up study the course of a large clinical sample of patients with eating disorders (EDs) who were treated with individual cognitive behaviour therapy. | Longitudinal (>5yr) | Relapse and diagnostic crossover | 7 |
| Chang et al., 2021 | Worldwide | Community (Young people 12+ and adults, Both Sexes) | 33 |  | To provide a comprehensive synthesis of literature on the early response phenomenon in the psychological treatment of EDs. | Review (Systematic) and Meta-Analysis | Remission, change in ED symptoms, associations between early response to ED treatment and outcomes | 112 |
| Colton et al., 2015 | Canada | Inpatient (Young People, Females) |  | 126 | To describe the longitudinal course of disturbed eating behaviour (DEB) and EDs in a cohort of women and girls with type 1 diabetes. | Longitudinal (>10yr) | Remission and recurrence | 42 |
| Custal et al., 2014 | Spain | Outpatient (Adult, Females) |  | 40 | To compare clinical, psychopathological and personality features between two samples of ED individuals: those with comorbid T1DM and those without (No-DM); and to identify differences in treatment outcomes between the groups. | Cross-sectional (Correlational) | Treatment outcome between individuals with eating disorders and diabetes and those without diabetes | 39 |
| Dalle Grave et al., 2009 | Italy | Inpatient (Adult, Females) |  | 152 | To test the prevalence, the associated features and the role on treatment outcome of self-induced vomiting in 152 ED patients consecutively admitted to an inpatient cognitive-behavioural treatment (CBT), based on the transdiagnostic CBT for ED. | Repeated Measure (without follow-up) | ED psychopathology using EDE; personality and general psychopathology at baseline and EOT | 74 |
| Dechartres et al., 2011 | France | Inpatient (Adult, Females) |  | 968 | To empirically classify phenotypes of eating disorders (ED) using latent class analysis (LCA), and to validate this classification based on clinical outcomes. | Modelling (Statistical - Diagnostic) & Retrospective Cohort Study | ED phenotype and outcome (SCL-90 score, HDRS score, treatment dropout, mortality) | 162 |
| Eielsen et al., 2021 | Norway | Outpatient (Adult, Both Sexes) |  | 62 | To investigate the 5- and 17-year outcome of adult patients with longstanding ED who were previously admitted to an inpatient ED unit. | Longitudinal (>10yr) | ED diagnoses and recovery, comorbid and general psychopathology, psychosocial functioning and quality of life at follow up. | 52 |
| Fernández-Aranda et al., 2021 | Spain | Day patient (Adult, Both Sexes) |  | 1199 | To examine how the duration of illness and other potential prognostic markers impacted on nonresponse and drop-out from treatment across different EDs subtypes. | Repeated Measure (without follow-up) | Full, partial or non-remission at EOT | 41 |
| Ferreira et al., 2017 | Brazil | Community (Adults, Both Sexes) |  | 60 | To improve current understanding of the course of depression and night eating after bariatric surgery in association with postoperative weight loss. | Repeated Measure (without follow-up) | Night eating symptoms, depressive symptoms | 86 |
| Fischer et al., 2014 | Switzerland | Outpatient (Adult, Both Sexes) |  | 41 | To evaluate the long-term efficacy (four years after treatment) of a short-term Cognitive-Behavioural Treatment (CBT) of Binge Eating Disorder. | Longitudinal (<5yr) | ED psychopathology, BMI, depression and anxiety symtpoms, general life satisfaction and general self-efficacy at follow-up. | 79 |
| Franko et al., 2018 | USA | Outpatient (Adult, Females) |  | 246 | To investigate predictors of long-term recovery from eating disorders 22 years after entry into a longitudinal study. | Longitudinal (>10yr) | Presence of an ED at follow-up (The Longitudinal Interval Follow-up Evaluation to assess ED recovery) | 61 |
| Genco et al., 2013 | Italy | Community (Adult, Both Sexes) |  | 50 | To evaluate the influence of double consecutive intragastric balloon treatment compared with single intragastric balloon treatment followed by diet, on four categories of EDNOS (grazing, emotional eating, sweet-eating and after-dinner grazing). | Repeated Measure (with follow-up) | Presence of EDNOS symptoms at baseline, 6 mths & 13 mths. | 85 |
| Glasofer et al., 2020 | USA | Inpatient (Mixed Cohort, Both Sexes) |  | 168 | To examine the association between clinical features at the time of hospital discharge and longer-term outcome among adolescents and adults with AN, the majority of whom had achieved weight restoration to a BMI within the normal range (18.5-24.9 kg/m2) | Longitudinal (>10yr) | Illness course (body mass index (BMI) and clinical impairment during the 5 years) and health maintenance (categories of weight and eating disorder symptom severity). | 93 |
| Glazer et al., 2019 | USA | Community (Adolescents, Females) |  | 9039 | To quantify eating disorder (ED) stability and diagnostic transition among a community-based sample of adolescents and young adult females. | Longitudinal (>10yr) | Number of years symptomatic; probability of maintaining symptoms; recovery; diagnostic crossover | 64 |
| Golden et al., 2021 | USA | Inpatient (Aged 12-24, Both Sexes) |  | 111 | To report the 1-year outcomes of a trial comparing higher with lower calorie refeeding. | RCT + Longitudinal Follow-up | BMI and ED psychopathology as measured by EDE-Q | 115 |
| Goldstein et al., 2011 | Australia | Outpatient (Young People, Both Sexes) |  | 26 | To present preliminary results of an open clinical trial of a day program for adolescents with AN. | Quasi-experimental (intervention) | Weight gain, AN symptoms, attitudes towards weight and shape | 65 |
| Gorrell et al., 2020 | USA | Outpatient (Young People, Both Sexes) |  | 110 | To examine reactivity in remission rates, relative to various conceptualizations of remission in a single RCT data set. | Modelling (Statistical) | Remission - various models | 31 |
| Grammer et al., 2021 | USA | Community (Adult, Both Sexes) |  | 8531 | To evaluate differences in probably diagnoses of EDs (AN, clinical/subthreshold BN, BED) and weight and shape concerns by sexual orientation and gender identity, and to investigate differences in ED chronicity and probable comorbid psychiatric diagnoses by sexual orientation and gender identity. | Cross-sectional survey | Weight and shape concerns, ED chronicity, psychiatric comorbidities | 107 |
| Guinhut et al., 2021 | France | Inpatient (Adult, Both Sexes) |  | 354 | To specify socio-demographic, anamnestic and clinical characteristics of AN patients hospitalised for extreme malnutrition, to identify types and prevalence of medical complications presented during their hospitalization for refeeding and the evolution of patient’s nutritional status. | Retrospective observational study | Weight gain, medical status | 150 |
| Hay et al., 2012 | Australia | Community (Adult, Females) |  | 828 | To investigate the persistence or otherwise of eating disorder symptoms, both behaviours and psychopathology, among a community sample over five years | Longitudinal (<5yr) | Remission of ED symptoms | 108 |
| Helverskov et al., 2010 | Denmark | Inpatient and Outpatient (Aged >13, Both Sexes) |  | 629 | To assess the 30-month outcome and predictors of outcome in a cohort of patients with any eating disorder (ED). | Longitudinal (<5yr) | Time to remission; predictors of remission; relapse; diagnostic crossover; and mortality. | 44 |
| Herpertz-Dahlmann et al., 2018 | Germany | Inpatient (Young People, Both Sexes) |  | 68 | To investigate the outcomes of patients with AN onset before the age of 14. | Longitudinal (>5yr) | Mortality, BMI, ED psychopathology, health-related quality of life,and comorbid psychopathology at follow-up. | 67 |
| Hilbert et al., 2020 | Worldwide | N/A | 114 | 8862 | To provide a comprehensive evaluation of the long-term effectiveness in diverse treatments for BED regarding a range of clinically relevant outcomes. | Meta-Analysis | Binge-eating episodes and abstinence, eating disorder and general psychopathology up to 12 months following treatment. | 77 |
| Hilbert et al., 2012 | USA | Outpatient (Adult, Both Sexes) |  | 90 | To examine the long-term efficacy of out-patient group cognitive–behavioural therapy (CBT) and group interpersonal psychotherapy (IPT) for binge eating disorder and to analyse predictors of long-term non-response. | Longitudinal (<5yr) post RCT | Remission assessed by ED psychopathology, general psychopathology, BMI, and healthcare utilisation. | 80 |
| Johnston et al., 2018 | Australia | Inpatient and Outpatient (Children and Adolescents, Females) |  | 175 | To examine the relationship between perfectionism at intake assessment and eating disorder symptoms in female youths with eating disorders across time from intake to 6 and 12 month review. | Cross-sectional (Correlational) and longitudinal follow-up | Scores on EDI-3 perfectionism subscale and remission status at follow-up | 26 |
| Kästner et al., 2019 | Worldwide | N/A | 68 |  | To investigate the differences in self-esteem between individuals with AN and healthy controls, or individuals with other eating disorders, and to examine self-esteem as an outcome, predictor, moderator, and mediator in AN treatment. | Systematic Review/ Meta-Analysis (combined) | Treatment outcome: remission; self-esteem | 118 |
| Keshishian et al., 2019 | USA | Inpatient (Adult, Females) |  | 176 | To examine the co-occurrence of depression and SUD in adult women who were recovered, relative to those who were not, at 22 years. | Longitudinal (>10yr) | Presence of comorbidities (major depressive disorder and substance use disorder) | 98 |
| La Mela et al., 2013 | Italy | Outpatient (Adult, Females) |  | 57 | To assess the roles of self-esteem, personality disorders (PDs), and dissociative experiences as treatment outcome predictors for CBT in ED patients. | Longitudinal (<5yr) | ED recovery | 103 |
| Lange et al., 2019 | Sweden | Outpatient (Adult, Both Sexes) |  | 56 | To compare long term outcome between childhood-onset Anorexia Nervosa (AN) and low-weight Avoidant/Restrictive Food Intake Disorder (ARFID) in regard to psychiatric diagnoses, social and occupational functioning. | Longitudinal (>10yr) | ED and general psychopathology, psychiatric comorbidity, mortality, social and occupational functioning at follow-up. | 24 |
| Le Grange et al., 2014 | USA | Outpatient (Young People, Both Sexes) |  | 79 | To report on relapse from full remission of AN and attainment of remission during a 4-year open follow-up period | Longitudinal (<5yr) | Incidence of relapse from full remission and new remission | 110 |
| Le Grange et al., 2014 | UK and Australia | Outpatient (Adult, Both Sexes) |  | 63 | To identify predictors and moderators of outcome at end of treatment (EOT) and 6- and 12-month follow-up for adults with AN treated with CBT-AN and SSCM. | Longitudinal (<5yr) | EDQoL, mental health and depressive symptoms at follow-up | 30 |
| Li et al., 2021 | Australia | Inpatient (Young People, Both Sexes) |  | 289 | To examine factors related to hospital length of stay (LOS), report referral on discharge, and hospital readmission, for children and adolescents (C&A) admitted to public hospitals for anorexia nervosa (AN) | Retrospective cohort study | Length of stay | 95 |
| Li et al., 2022 | Worldwide | N/A | 3; 9 |  | To identify existing interventions for people with ED and comorbid autism spectrum condition (ASC), critically review evidence of their clinical effectiveness and cost-effectiveness, and review the impact of ASC comorbidity on ED clinical outcomes. | Review (systematic) | ED clinical outcomes (physical, psychopathological, BMI) | 104 |
| Linardon, 2018 | Worldwide | N/A | 39 |  | To estimate the prevalence of patients with BED who achieved binge eating abstinence following psychological or behavioural treatments. | Meta-Analysis | Abstinence from binge eating following treatment. | 72 |
| Linardon et al., 2018 | Worldwide | N/A | 45 |  | To (a) estimate the prevalence of patients who abstain from binge eating and/or purging following all psychological treatments for BN, and (b) test whether these abstinence estimates are moderated by the type of treatment modality delivered, the definition of abstinence applied, and trial quality. | Meta-Analysis | Abstinence from purging post BN treatment | 161 |
| Linardon et al., 2017 | Australia | N/A | 65 |  | To synthesise the literature on predictors, moderators, and mediators of outcome following Fairburn's Cognitive Behavoural Therapy for eating disorders. | Review (Systematic) | Predictors, moderators, and mediators of treatment outcome | 22 |
| Lydecker et al., 2021 | USA | Outpatient (Adult, Both Sexes) |  | 636 | To determine whether psychiatric comorbidity predicted or moderated BED treatment outcomes. | RCT and Repeated Measures (without follow-up) | ED psychopathology, psychiatric comorbidity, percent weight loss. | 51 |
| Mason et al., 2017 | USA | Outpatient (Adult, Both Sexes) |  | 171 | To examine baseline and longitudinal associations between eating-related and psychosocial variables and dimensions of weight QOL. | Repeated Measure (without follow-up) | BMI, QoL, ED psychopathology, depressive symptomatology, self-esteem at baseline, midpoint and EOT. | 89 |
| McClelland et al., 2020 | UK | Inpatient and Outpatient (Young People, Both Sexes) |  | 322 | To a) estimate the proportion of patients in a CAEDS who use mental health services as young adults, (b) to delineate service utilisation following treatment in CAEDSs and (c) to identify factors in CAEDSs that predict service utilisation in young adulthood. | Longitudinal (>5yr) | Mental health service utilisation at follow-up | 114 |
| Micali et al., 2015 | UK | Community (Adolescents, Both Sexes) |  | 5069 | To investigate whether anorexia nervosa (AN), bulimia nervosa (BN), binge eating disorder (BED), and other specified feeding and eating disorders (OSFED), including purging disorder (PD), subthreshold BN, and BED at ages 14 and 16 years, are prospectively associated with later depression, anxiety disorders, alcohol and substance use, and self-harm. | Longitudinal (<5yr) | ED symptomatology/diagnosis, drug and alcohol use, anxiety and depressive symptoms, self-harm and BMI at follow-up. | 101 |
| Munsch et al., 2012 | Switzerland | Outpatient (Adult, Both Sexes) |  | 80 | To assess the long-term efficacy of Cognitive-Behavioural Treatment (CBT) and Behavioural Weight-Loss-Treatment (BWLT) in patients with binge eating disorder (BED) and to identify potential predictors of long-term treatment success. | Longitudinal (>5yr) post RCT | ED psychopathology, BMI, negative affect, therapeutic process and contentment with therapy at follow-up. | 82 |
| Murray et al., 2022 | Worldwide | N/A | 20 |  | To review of the efficacy of stereotactic ablative neurosurgery, DBS, TMS and tDCS, exclusively in the context of AN | Review (Systematic) | Weight normalisation and psychological symptom indices post-treatment. | 163 |
| O'Brien et al., 2017 | USA | Community (Adult, Females) |  | 47,759 | To study predictors of self-reported eating disorders and associations with later health events | Retrospective cohort study | ED symptomatology, BMI, cigarette smoking, depression, infertility. | 99 |
| Opozda et al., 2016 | Worldwide | N/A | 23 |  | To systematically review and compare the literature on presurgery to postsurgery changes in the following problematic and disordered eating behaviours after gastric bypass, banding and sleeve: binge eating disorder and associated behaviours (e.g. binge episodes and uncontrolled eating); bulimia nervosa and associated behaviours; emotional eating; night eating syndrome; and grazing. | Review (Systematic) | Changes to problematic and disordered eating behaviours post weight-loss surgery | 84 |
| Paul et al., 2015 | The Netherland | Community (Adult, Both Sexes) |  | 128 | To examine the added value of pre-operative cognitive behavioural therapy (CBT) focused on modification of thoughts and behaviours in terms of eating behaviour and physical exercise as well as preparation for surgery and postoperative lifestyle. | Methodology paper | Weight loss, eating behavior, eating disorders, depression, quality of life and psychological distress pre-post treatment and 1, 3, 5 year follow-up. | 90 |
| Quadflieg et al., 2019 | Germany | Inpatient (Aged >15, Both Sexes) |  | 2033 | To assess the long-term outcome and identify outcome predictors in a very large sample of inpatients treated for bulimia nervosa (BN). | Longitudinal (>10yr) | Eating disorder psychopathology and psychiatric comorbidity at follow-up | 45 |
| Radunz et al., 2020 | Worldwide | N/A | 31 |  | To examine the contribution of duration to treatment outcome for eating disorders. | Systematic Review/ Meta-Analysis (combined) | Recovery, eating disorder psychopathology, weight gain | 94 |
| Ricca et al., 2010 | Italy | Outpatient (Aged >15, Females) |  | 103 | To evaluate the effects of individual CBT in outpatients with threshold and subthreshold AN at EOT and 3 year follow-up; and to identify possible predictors of outcome. | Longitudinal (<5yr) | ED psychopathology using EDE-Q; body uneasiness and general psychopathology at baseline, EOT and 3 year follow-up. | 57 |
| Rigaud et al., 2011 | France | Inpatient (Adult, Both Sexes) |  | 484 | The aim of our present study was to determine the outcome of 484 purely AN adult patients followed-up for 13 years, and to identify prognostic factors from those recorded at the start of follow-up. | Longitudinal (>10yr) | Long-term outcome: full recovery, 'relatively' good outcome; persistent illness ('bad' or 'severe'); death. | 20 |
| Sansfaçon et al., 2020 | Worldwide | N/A | 42 |  | To quantify the association between pretreatment motivation and posttreatment changes in eating disorder symptomology. | Systematic Review/ Meta-Analysis (combined) | ED symptomatology post-treatment | 120 |
| Santomauro et al. 2021 | Worldwide | N/A | 54 |  | To estimate the prevalence and burden of binge-eating disorder and OSFED globally. | Review (other) | Prevalence and burden of BED and OSFED globally, disability-adjusted life-year (DALY) | 147 |
| Shapiro et al., 2010 | USA | Community (Adult, Females) |  | 31 | To examine a text-messaging program for self-monitoring symptoms of bulimia nervosa (BN) within the context of cognitive-behavioural therapy (CBT). | Quasi-experimental (intervention) | Self-reported binge and purge episodes | 126 |
| Sollid et al., 2021 | Denmark | Community (Adult, Females) |  | 122 | To investigate the occurrence of perinatal ED relapse and obstetric and postpartum outcome in women with at least 6-month ED remission before pregnancy. | Cross-sectional | Relapse, clinical outcomes (hyperemesis gravidarum, body image, psychiatric disorders, postpartum depression) | 106 |
| Steinhausen et al., 2009 | Worldwide | N/A | 79 | 5653 | To broadly assess the outcome of bulimia nervosa, effect variables, and prognostic factors. | Systematic Review/ Meta-Analysis (combined) | Clinical outcome of bulimia nervosa: recovery, improvement, chronicity, crossover to another eating disorder, mortality, and comorbid psychiatric disorders at outcome. | 63 |
| Stice et al., 2013 | USA | Community (Children, Young females) |  | 496 | To examine the lifetime prevalence and annual incidence of DSM-5 eating disorders by age 20 and the peak periods of risk for onset of these disorders (Aim 1); whether individuals with DSM-5 eating disorders show impairment relative to eating disorder-free youth in terms of functioning, emotional distress, suicidality, mental health treatment, and an unhealthy body mass (Aim 2); and the episode duration, remission rates, recurrence rates, diagnostic progression, and diagnostic crossover for DSM-5 eating disorders (Aim 3). | Longitudinal (>5yr) prospective cohort study | ED psychopathology, functional impairment, emotional distress, suicidality mental health service utilisation and BMI at baseline and consecutive follow-up. | 27 |
| Stice et al., 2009 | USA | Community (Adolescents, Females) |  | 496 | To examine the (a) lifetime prevalence and incidence of threshold and subthreshold AN, BN, BED, and PD by age 20 years; (b) peak periods of risk for onset of these disorders; (c) duration and relapse rates for these eating disturbances; and (d) progression from subthreshold to threshold diagnoses of the same disorder and crossover from one eating disorder to another. | Longitudinal (>5yr) | ED course and diagnostic crossover | 33 |
| Støving et al., 2020 | Denmark | Inpatient and Outpatient (All Ages, Both Sexes) |  | 7505 | To explore time-trends in treatment modes of patients with free and equal access to health services. | Longitudinal (<5yr) | Per patient number of hospital admissions, cumulated number of days of hospitalization and number of outpatient visits during the first 5 years after initial diagnosis. | 113 |
| Strandjord et al., 2015 | USA | Inpatient (Young People, Both Sexes) |  | 244 | To compare hospitalized ARFID and AN patients at presentation, during hospitalization, and 1 year after discharge. | Review (Other) | Remission | 25 |
| Tasca et al., 2012 | Canada | Inpatient and Outpatient (All Ages, Both Sexes) |  | 837 | To characterise a tertiary care treatment-seeking sample and assess post-treatment remission and completion rates for purging disorder (PD). | Cross-sectional cohort study | Eating disorder-specific and general psychopathology; post treatment remission and completion | 91 |
| Thompson-Brenner et al., 2021 | USA | Inpatient (All Ages, Gender not reported) |  | 345 | To examine the effect of the Renfrew Unified Treatment for ED and Comorbidity (UT) implementation across five years of treatment delivery. | RCT | ED symptom severity (using EDE-Q), Depressive symptoms, experiential avoidance, anxiety sensitivity, clinical diagnosis | 117 |
| Tomba et al., 2017 | Italy | Outpatient (Adult, Females) |  | 185 | To examine whether dimensions of psychological well-being in ED out-patients change after CBT-based and nutritional rehabilitation treatment and whether any observed gains in remitted patients reach optimal levels found in matched healthy controls. | Quasi-experimental (intervention) | Psychological well-being | 43 |
| Villarejo et al., 2012 | Spain | Inpatient (Adult, Females) |  | 1383 | To examine the lifetime prevalence of obesity rate in eating disorders (ED) subtypes and to examine whether there have been temporal changes among the last 10 years and to explore clinical differences between ED with and without lifetime obesity. | Retrospective cohort study | Lifetime presence of obesity; ED symptoms, general psychopathology and personality. | 81 |
| Wadden et al., 2011 | USA | Community (Adult, Both Sexes) |  | 59 | To determine whether bariatric surgery candidates who were diagnosed with BED would lose significantly less weight 1-year post-surgery than would patients who were free of this condition. | Longitudinal (<5yr) | Body weight, frequency of binge eating and cardiovascular disease risk. | 87 |
| Wade et al., 2021 | UK | Outpatient (Adult, Gender not reported) |  | 187 | To better understand those patients with anorexia nervosa who do not show early response to treatment and are likely to have poorer outcome. | Repeated Measure (with follow-up) | ED psychopathology using EDE-Q, depression and anxiety symptomatology, functional impairment. | 47 |
| Walker et al., 2021 | USA | Outpatient (All Ages, Both Sexes) |  | 210 | To replicate findings from randomized controlled research trials and inpatient samples, identifying treatment outcome predictors in a transdiagnostic ED IOP sample. | Repeated Measure (without follow-up) | ED psychopathology; rapid response to treatment; individual, interpersonal, social, and overall well-being; and physical status. | 37 |
| Wild et al., 2016 | Germany | Outpatient (Adult, Females) |  | 169 | To determine predictors of BMI and recovery for outpatients with anorexia nervosa (AN). | RCT | BMI, ED psychopathology, disorder severity, psychiatric comorbidity, self-esteem and health-related quality of life at 1-year follow-up | 55 |
| Winkler, 2017 | Denmark | Inpatient and Outpatient (All Ages, Both Sexes) | 7 | 383, 998, 113 | To report on and determine predictors of HRQoL in ED; to report ED pathology and determine predictors of clinical outcome; to evaluate the correlation between patient-reported outcome and clinical characteristics; to examine mortality rates; and to investigate the association between body composition and menstrual status. | Cross sectional cohort study; meta-analysis; retrospective cohort study | HRQoL; EDI-2 symptom score; mortality; resumption of menses; body fat %; BMI. | 109 |
| Zerwas et al., 2013 | North America & Europe | Inpatient and Outpatient (Aged >13, Females) |  | 680 | To examine the association between prognostic factors—eating disorder features, personality traits, and psychiatric comorbidity—and likelihood of recovery in a large sample of women with AN | Longitudinal (>5yr) | Full recovery from AN (at least one year with no symptoms) | 56 |
| Kelly et al., 2014 | Canada | Inpatient and Outpatient (Adult, Females) |  | 252 | To investigate the relative contributions of self-compassion, fear of self-compassion, and self-esteem in eating disorder pathology between undergraduate students and females diagnosed with an eating disorder entering treatment. | Cross-sectional cohort study | EDE-Q, self-compassion, fear of self-compassion, self-esteem | 119 |
| Relapse prevention programs | | | | | | | | |
| Anastasiadou et al., 2018 | Spain | N/A | 15 |  | To systematically review the existing evidence of mobile health (mHealth) tools for the treatment of eating disorders (ED). | Review (Systematic) | Efficacy of mobile health (mHealth) tools for the treatment of eating disorders (ED) | 128 |
| Bauer et al., 2012 | Germany | Inpatient (Adult, Females) |  | 165 | To investigate the efficacy of a technology enhanced program to support patients after their discharge from inpatient treatment against a control condition (treatment as usual; TAU). | RCT | Partial remission (or better) at 8 months follow up; abstinence from bulimic behaviours | 127 |
| Berends et al., 2016 | The Netherlands | N/A |  | 83 | To examine the rate, timing and predictors of relapse of patients who were treated with the Guideline Relapse Prevention for AN program. | Cohort | Rate of relapse, timing of relapse, factors associated with relapse | 36 |
| Fichter et al., 2013 | Germany | Inpatient (Adult, Females) |  | 258 | To evaluate the efficacy of a 9-month internet-based intervention program for AN, compared to a group of AN patients receiving treatment as usual (TAU) following discharge from inpatient therapy. | RCT | Efficacy of VIA in relapse prevention measured primarily via change in BMI | 19 |
| Gulec et al., 2014 | Hungary | Outpatient (Adult, Females) |  | 105 | To investigate the preliminary use and acceptance of an internet-based post-care support program in maintaining and/or enhancing treatment gains against a wait-list TAU control condition. | RCT | Eating disorder related attitudes via Eating Disorder Examination Questionnaire (EDE-Q) | 125 |
| Jacobi et al., 2017 | Germany | Inpatient (Adult, Females) |  | 253 | To determine the efficacy of an internet-based aftercare program in maintaining treatment gains for women with BN following inpatient treatment. | RCT | Abstinence from any core BN symptoms | 124 |
| Mortality | | | | | | | | |
| Ackard et al., 2014 | US | Inpatient (All ages, Females) |  | 219 | To (1) discern sociodemographic and clinical differences, (2) determine outcome rates, and (3) identify predictors of poor outcome including death. | Longitudinal (>5yr) | Clinical outcome including mortality; predictors of poor outcome | 28 |
| Arcelus et al., 2011 | Worldwide | N/A | 36 |  | To systematically compile and analyse the mortality rates in individuals with anorexia nervosa (AN), bulimia nervosa (BN), and eating disorder not otherwise specified (EDNOS). | Meta-Analysis | Mortality | 4 |
| Button et al., 2009 | UK | Outpatient (Adult, Both Sexes) |  | 1892 | To investigate mortality across the spectrum of eating disorders; and to explore whether any clinical and demographic factors may be associated with increased risk. | Longitudinal (>5yr) | Mortality, predictors of mortality | 129 |
| Chesney et al., 2014 | Worldwide | N/A | 20 |  | To explore the risks of all-cause and suicide mortality in major mental disorders. | Review (Systematic) | Mortality, cause of death | 150 |
| Crowet al. 2014 | US | Outpatient (Adult, Both Sexes) |  | 13,103 | To determine whether anorexia nervosa, bulimia nervosa, and eating disorder not otherwise specified are associated with increased all-cause mortality or suicide mortality. | Longitudinal (>10yr) | Mortality; cause of death | 142 |
| Fichter et al., 2021 | Germany | Inpatient (Adult, Both Sexes) |  | 5484 | To report on the mortality of DSM-IV eating disorders and predictors of premature death in males compared to females after inpatient treatment. | Longitudinal (>10yr); Case-control analytic study | Mortality | 2 |
| Fichter et al., 2016 | Germany | Inpatient (Adult, Both Sexes) |  | 5839 | To report on long-term mortality in anorexia nervosa (AN), bulimia nervosa (BN), binge eating disorder (BED), and eating disorder not otherwise specified (ED-NOS), causes of death, and predictors of early death. | Longitudinal (2-20+ years) | Mortality and cause of death | 135 |
| Franko et al., 2013 | US | Outpatient (Aged >12, Females) |  | 246 | To examine standardized mortality ratios at two longitudinal points and also at varying years of duration of the illness, to see whether there is a peak period for death in eating disorders; and to examine factors that might increase the vulnerability to premature death. | Longitudinal (>10yr) | Mortality, predictors of mortality | 49 |
| Guinhut et al., 2021 | France | Inpatient (Aged >15, Both Sexes) |  | 384 | To estimate standardized mortality ratio (SMR), investigate predictive factors of mortality and causes of death among a sample of patients with AN admitted to a specialized clinical nutrition unit (CNU) because of extremely severe malnutrition. | Longitudinal (>5yr) | Mortality, SMR | 112 |
| Hoang et al., 2014 | UK | Inpatient (All Ages, Both Sexes) |  | 34,166 | To calculate mortality of people with eating disorders (ED) in England, relative to that of people of the same age and sex, between 2001 and 2009, with a focus on adolescents and young adults (15–24 years), and older adults (25–44 years). | Modelling (Statistical) | Mortality, SMR, cause of death | 111 |
| Huas et al., 2011 | France | Inpatient (Adult, Females) |  | 601 | To establish mortality rates and identify predictors in a large sample of adults through a 10-year post in-patient treatment follow-up. | Longitudinal (>5yr) | Crude and standardised mortality | 68 |
| Iwajomo et al., 2021 | Canada | Inpatient and Outpatient (All Ages, Both Sexes) |  | 19,041 | To investigate all-cause mortality in a population-based cohort of individuals who received hospital-based care for an eating disorder (anorexia nervosa, bulimia nervosa or eating disorder not otherwise specified). | Modelling (Statistical) | Mortality, SMR | 133 |
| Keshaviah et al., 2014 | Worldwide | N/A | 40 |  | To re-estimate mortality after methodological corrections and to identify predictors of mortality. | Meta-Analysis | Mortality, predictors of mortality | 130 |
| Keski-Rahkonen, 2021 | Worldwide | N/A | N/A |  | To provide an overview of prevalence, course, comorbidity, mortality, and risk factors of BED based on major studies published in 2018-2021. | Review (narrative) | Mortality, prevalence of BED, comorbidities | 76 |
| Mellentin et al., 2022 | Denmark | Community (All Ages, Both Sexes) |  | 20,759 | To examine the association of alcohol use disorders and other SUDs with mortality in AN, BN, unspecified ED compared with matched control subjects. | Retrospective cohort | Alcohol use disorders and other SUD with mortality | 144 |
| Naghavi et al., 2017 | Worldwide | N/A |  | 264 | To provide a peer-reviewed, comprehensive, and annual assessment of mortality by age, sex, cause, and location. | Modelling (Statistical) | Mortality and cause of death | 146 |
| Quadflieg et al., 2019 | Germany | Inpatient (Adult, Males) |  | 338 | To report on the long-term mortality of eating disorders in male inpatients. | Longitudinal (>10yr) | Mortality | 45 |
| Rosling et al., 2011 | Sweden | Inpatient (Adult, Females) |  | 201 | To study excess mortality, causes of death, and co-morbidity in patients with eating disorder (ED), treated in a Swedish specialist facility. | Longitudinal (>10yr) | Mortality and cause of death, comorbidity | 141 |
| Suokas et al., 2013 | Finland | Inpatient and Outpatient (Adult, Both Sexes) |  | 2450 | To investigate mortality rates and mortality patterns in the course of follow-up in patients treated in a specialised eating disorder unit for AN, BN and BED in Finland. | Longitudinal (>10yr) | Mortality | 136 |
| van Hoeken et al., 2020 | Worldwide | N/A | N/A |  | To review the recent literature on the burden of eating disorders in terms of mortality, disability, quality of life, economic cost, and family burden, compared with people without an eating disorder. | Review (Systematic) | Mortality, disability, costs, quality of life, and family burden | 3 |
| Wentz et al., 2009 | Sweden | Community (Adolescents, Both Sexes) |  | 51 | To study prospectively the long-term outcome and prognostic factors in a representative sample of people with teenage-onset anorexia nervosa. | Longitudinal (>10yr) | Mortality and presence of ED | 59 |
| Winkler et al., 2015 | Denmark | Outpatient (Adult, Both Sexes) |  | 998 | To establish mortality rates in a sample of ED patients with a mean follow-up of 12 years. | Longitudinal (>10yr) | Mortality; SMR | 58 |
| Zerwas et al., 2015 | Denmark | Inpatient and Outpatient (All ages, Both Sexes) |  | 966 | To characterise the incidence rates and cumulative incidence of anorexia nervosa (AN), bulimia nervosa (BN), and eating disorder not otherwise specified (EDNOS), and examine associations among eating disorder diagnoses, suicide attempts, and mortality. | Retrospective cohort | ED incidence rate, cumulative incidence, death and suicide rates | 143 |
